# Need for Inclusive Consideration of Transgender and Gender Diverse People in E-Health Services: A Systematic Review

**DOI:** 10.3390/jcm11041090

**Published:** 2022-02-18

**Authors:** Janis Renner, Lars Täuber, Timo O. Nieder

**Affiliations:** University Medical Center Hamburg-Eppendorf, Institute for Sex Research, Sexual Medicine and Forensic Psychiatry, Martinistr. 52, 20246 Hamburg, Germany; l.taeuber@uke.de (L.T.); tnieder@uke.de (T.O.N.)

**Keywords:** e-health, digital health, transgender, gender diverse, trans health care, LGBT, systematic review

## Abstract

Many transgender and gender diverse (TGD) people use the internet to find ways out of isolation, network, and share information on health-related topics. Thus, e-health services could reduce the health burden of TGD people and facilitate access to health care. Following the PRISMA guidelines, we conducted a systematic review on e-health approaches that could improve trans health care (i.e., services directly for TGD people or training programs for health care professionals, HCPs) and their effectiveness, acceptability, and feasibility. We searched PubMed, Web of Science, and PubPsych databases for publications from January 2000 to June 2021 with final updates before publication. The systematic review identified e-health services across 27 studies from 8 different countries. Few studies evaluated e-health services exclusively for TGD people. However, use of an e-health service was found to be effective and beneficial: TGD people improved in health-related outcomes, and HCPs improved in professional expertise. Service users find e-health services helpful and easy to integrate into their daily lives. Recommendations for further development of e-health services in trans health care are provided. In the future, given the rapidly evolving e-health research and care field, new treatment approaches for TGD people should be subject to ongoing evaluation and development.

## 1. Introduction

The internet has long been considered an important medium, especially for young LGBTQIA+ (short for lesbian, gay, bisexual, transgender, queer, intersex, and asexual, with the + intended to capture all identities and expressions) populations who use the internet daily to access information, communicate, and consume and share content through various platforms [1]. As a subgroup of LGBTQIA+ individuals, for transgender and gender diverse (abbreviated to TGD; in accordance with the terminology chapter of the WPATH Standards of Care V8 (SOC 8, currently being developed) TGD people refers to individuals whose gender identity or expression differs from the gender socially attributed to their assigned sex at birth) people, the internet provides an opportunity to connect with peers. Trans-affirming online experiences prove to be a safe space for TGD youth, allowing for safety, support, and community belonging, as well as an escape from stigma and violence [2]. This is all the more important given the well-known high risk of TGD people for depression, anxiety, and somatization [3,4,5,6]. TGD people diagnosed with gender dysphoria often report impaired mental health, with studies suggesting that gender-affirming medical interventions (e.g., hormone therapy, genital surgery) alleviate psychological distress in the face of increasing congruence between self-image and physical appearance [7,8]. Significant contributions to impaired mental health are made by stigmatization towards TGD people at structural (e.g., societal norms, laws, and policies), interpersonal (e.g., everyday interactions), and individual (e.g., beliefs and behaviors) levels [9]. According to social-ecological models, stigma has a lasting negative impact on health, which expresses itself structurally—among other things—in barriers to accessing health care [9,10]. In particular, this affects TGD people living in remote, rural, or suburban areas where connections to peers and adequate health care are often lacking or absent [11].

Better access to trans health care and training for health care professionals (HCPs) are seen as two promising interventions to reduce stigma against TGD people [9]. To overcome access barriers such as long waiting times or travel distances to specialty clinics, internet-based interventions (e.g., mental health services such as counseling or psychotherapy) are increasingly being trialed and their effectiveness empirically tested to assess whether they can usefully supplement the range of services offered by HCPs [12]. Expectedly, internet interventions could reach TGD people well in their lived realities (e.g., aiming at an initial relief of psychological distress or to connect with medical experts for gender-affirming health care), particularly those in underserved remote, rural, or suburban areas [11,13]. At the same time, implementing TGD-related training curricula, supervision, and consultations for HCPs online could advance professionalization in trans health care and further reduce barriers resulting from stigma [14,15,16]. The need to train HCPs extends to all health care disciplines, from primary care to nursing, whose practice-based training curricula should enable them to work from a TGD-informed standpoint [17,18,19,20].

E-health is a rapidly growing phenomenon that encompasses innovative approaches with various sub-concepts using information and communication technologies (ICT), aimed at improving the health of their users [21]. In this review, we use e-health as an umbrella term. Various e-health platforms provide self-paced modularized, task-based, or game-based engagement with health-related issues, such as depression, stress, or alcohol use reduction [22,23,24,25]. Furthermore, telehealth is about bridging distances so that HCPs themselves actively provide health care services using ICT. Internet- and computer-based interventions with a telehealth approach for anxiety and depression have been the subject of several systematic reviews and meta-analyses [26,27,28]. Telehealth encompasses a wider range of services with health-related activities such as complementary preventive care [29]. Due to their use of video consultation as an ICT, telemedicine and telehealth are often used interchangeably [29]. However, telemedicine is a subset of telehealth and focuses on health services provided by HCPs [29]. In the case of trans health care, telemedicine treatment may be an endocrinology consultation via video consultation [30]. Mobile health, or mHealth for short, refers to a growing number of mobile technologies such as apps for health information and services for a variety of application fields from stress management to clinically relevant conditions [31,32,33,34,35]. E-health also includes not only purely digital services, but also the integration of internet interventions and regular face-to-face treatment, so-called blended care [36], a concept that is used primarily in the field of mental health and whose effectiveness has been demonstrated in systematic reviews [37]. Other less narrowly defined online approaches such as online self-help, online coaching, or social media health approaches seem rather unconventional for health care but are very present in online forums or social media and complement the e-health concept [38]. Online or SMS notifications, discussion forums, or chats as simple professional or community-based online intervention approaches, for example, support existing e-health approaches [33,38,39]. It should be noted that the dynamic nature of the e-health umbrella term makes it difficult to delineate concepts, also with regard to the ongoing development and programming of new applications [12,40,41]. See Table 1 for an overview of e-health applications.

For clinical issues, such as the treatment of depression, research on the cost effectiveness of e-health approaches is already advanced [28]. Thus, these fields of application permit statements for the cost effectiveness in health care through the expansion of e-health services. Comparable studies for trans health care are lacking. However, e-health pilots for TGD have begun, providing indications of needs, feasibility, and acceptance [42,43,44,45,46]. At this stage, research indicates that, in general, e-health interventions can be helpful even for severely burdened patients [34]. The same could be true for TGD people at risk due to multiple stigmatization [3,4,5,6,9,11]. Often, TGD people are included in LGBTQIA+ studies regardless of context, rather than TGD-related projects being specifically designed. However, by subsuming TGD people into the LGBTQIA+ community, their specific needs may remain unaddressed. E-health services that aim to reduce stigma and improve trans health care are nonetheless likely to be investigated in both TGD-only studies and studies with LGBTQIA+ samples. In addition, e-health services to educate HCPs could also focus on these aims. Given the novelty of e-health in trans health care, it is essential to consider and evaluate the diversity of e-health approaches (i.e., different e-health sub-concepts) and sample compositions (i.e., TGD, LGBTQIA+ community members, or HCPs) in a systematic review so as not to exclude relevant studies.

Systematic reviews exist for e-health interventions for young LGBTQIA+ people [47], e-health interventions purely for men who have sex with men (MSM) [48], mHealth approaches used for HIV prevention for adolescent TGD people [49], and telemedicine for gender-affirming care [50]. To our knowledge, there is no systematic review to date that addresses e-health for TGD people in all its sub-concepts and examines effectiveness, acceptability, and feasibility in the context of TGD-only samples, LGBTQIA+ samples, and HCP samples. Based on this, we analyzed what e-health services exist for TGD people, and whether and how they improve trans health care, and thus support the health and quality of life of TGD people. Therefore, our research questions are as follows:

(1). Which e-health services have been empirically tested that improve the health of TGD people directly through treatments for TGD people or indirectly through qualifying HCPs?
(2). How effective are e-health services in improving the health of TGD people?
(3). How acceptable and feasible are e-health services for trans health care?

## 2. Materials and Methods

### 2.1. Protocol and Registration

The protocol for this systematic review was registered in PROSPERO (Prospective Register of Systematic Reviews) with ID CRD42021258870. For the article, we followed the PRISMA guidelines (Preferred Reporting Items for Systematic Reviews and Meta-Analyses) [51].

### 2.2. Eligibility Criteria

#### 2.2.1. Participants

The main target group for this systematic review was TGD people regardless of age. We assumed that few e-health approaches exist purely for TGD people. For this reason, we also accepted LGBTQIA+ e-health studies as long as they intentionally demonstrated value and outcomes for TGD people. Therefore, we excluded LGBTQIA+ studies if their sample did not include TGD people. Finally, we included studies that analyzed interventions designed for HCPs with the goal of improving health care and increasing knowledge about TGD people. This related to e-health services for HCPs with LGBTQIA+ training content, which required ensuring that the training service provided TGD health content for inclusion in the systematic review. The selection process from the 1150 articles initially found via the literature databases to the final 27 included studies can be seen in the flow diagram (Supplementary File S1).

#### 2.2.2. Interventions

We included any intervention using e-health technologies that aimed to improve the general and mental health or quality of life of TGD people. Specifically, we refer to e-health services designed exclusively for TGD people, for LGBTQIA+ people including TGD people, or for HCPs to enhance their expertise in trans health care. Regarding e-health, we were interested in the e-health sub-concepts presented in the introduction and Table 1. The e-health services we considered therefore cover a wide range of intervention types. For ease of reading, we use the term e-health to refer to all interventions, which aim to improve the health of TGD people through electronic, digital services. Regarding eligibility criteria, we included only studies of actually implemented e-health services (i.e., interventional or observational studies) to draw conclusions about the effectiveness, acceptability, and feasibility of e-health approaches in trans health care. Studies that simply outlined the presumed benefits of e-health and did not evaluate interventions were excluded from the systematic review.

#### 2.2.3. Studies

Empirical studies were included in the review regardless of their methods used. Quantitative, qualitative, and mixed methods studies were eligible and could be evaluated. We considered studies from 2000 onward and chose this cut-off because precise definitions of e-health were just beginning to be published in the early 2000s [21] and technologically advanced e-health approaches did not play a role in trans health care before this century. We did not set a geographic focus for the review (i.e., e-health services from all countries) but only considered peer-reviewed publications if they were written in English or German given our own language skills. Unpublished theses were only included by manually checking the reference lists of hits found in the literature database search. All studies to be included should include keywords or descriptions for e-health approaches for TGD people. We excluded both original research on e-health approaches that did not address TGD people, and study protocols, reviews, meta-analyses, commentaries, and editorial pieces. Because we were interested in papers on e-health (i.e., interventional or observational studies), we also did not include guidelines or manuals in our search strategy.

#### 2.2.4. Outcomes

According to the research questions, study-specific measurements of the overall effectiveness, acceptability, or feasibility of an e-health service were explored and described. Studies on e-health services in trans health care are a novelty, so we expected heterogeneity in outcomes. Across the studies, we assumed very diverse objectives for e-health services, so outcomes are likely to be study-specific to the particular e-health service. E-health services do not commit to a single application area in trans health care (e.g., mental health counseling, HIV/STI care). Thus, we pooled any studies describing e-health services that provide knowledge, assistance, or support to improve general and mental health and quality of life. E-health approaches could still be in the evaluation and further development phase but should not be in an earlier development phase that does not guarantee statements about outcomes of interest to the review.

### 2.3. Search Strategy

For the systematic review objectives, we considered three concepts in the search strategy that were linked with the Boolean operator “AND”: (1) sexual and gender minorities, (2) transgender, and (3) e-health services. For each of the three concepts, we formulated a variety of keywords to cover the range of concepts. “Transgender” belongs to the concept of “sexual and gender minorities” in terms of content, but we linked the concepts with “AND” to ensure that the hits we found covered e-health studies that addressed TGD people. An unfocused search would have been ineffective [52]. We searched the PubMed, Web of Science, and PubPsych databases for the period 2000 to June 2021, with final updates to include possible additional papers just before manuscript submission. Because of the novelty of e-health in trans health care, we designed the search strategy with a focus on high specificity, meaning false positive hits were accepted to avoid exclusion of relevant studies. E-health-relevant terms included the wide range of services that broadly represented online support for TGD people. In Supplementary File S3, we list the full search strategy. At the same time, the search strategy was deposited in PROSPERO. In addition to the main search via the literature databases, we scanned the reference lists of the retrieved studies for relevant papers and, for excluded study protocols, checked whether the results of the protocoled studies had already been published.

### 2.4. Screening

The process of independent screening and review of included studies was guided by Muka et al. [52]. Found hits via the literature database search were uploaded by J.R. to Rayyan [53]. Rayyan is a free web and mobile app that simplifies screening in a user-friendly way via easy-to-use decision buttons and customizable labels and comments. Blinded screening is also possible and was used to ensure independence of reviewer screening judgements and reproducibility of the process. J.R. shared the dataset with L.T. For duplicate identification, Rayyan provides automatic detection, but deduplication was conducted manually by J.R. and L.T. to ensure correctness. For the title–abstract screen, both J.R. and L.T. screened the titles and abstracts independently. Disagreements in the selection process were resolved by both through verbal dialogue. Following the title–abstract screen, we searched for the full texts that passed the initial screening and stored them in Mendeley. Uploading full texts is not possible in Rayyan. At the same time, Rayyan ensures transparent screening, which is why we implemented the use of Rayyan and additionally a literature management program based on recommendations [52]. Again blinded, J.R. and L.T. independently screened the full texts for fit to the systematic review. The procedure for discrepancies with the studies to be included after full-text screening was similar to the title–abstract screen phase (i.e., review of inclusion and exclusion criteria, verbal dialogue between J.R. and L.T. in case of an unclear fit of studies, and final inclusion of appropriate studies). For final non-matches, T.O.N. was consulted as an independent third party.

### 2.5. Data Extraction

As recommended, a spreadsheet was used for data extraction, which we designed according to our review objectives [52]. The table included the following sections: author(s), study title, publication year, intervention, study design, main variables, sample composition with age (mean and range) and proportion of TGD people, location of study, e-health approach used including platform chosen, research questions and methodology, quantitative or qualitative findings, summary of findings relevant to the systematic review, and final assessment. L.T. cross-checked the data. An overview can be found in Supplementary File S2.

### 2.6. Critical Appraisal

The different study types led to the use of different assessment tools to ensure that studies were adequately assessed with a validated instrument specific to the study type. For randomized controlled trials, the revised version of the Cochrane Risk of Bias Assessment Tool RoB 2 was used [54]. Quantitative non-randomized studies were assessed using ROBINS-I (short for “Risk of Bias In Non-randomized Studies—of Interventions”) [55]. For qualitative studies, we used an adapted version of the Critical Appraisal Skills Programme (CASP) tool [56]. We used the Mixed Methods Appraisal Tool (MMAT) [57] conditionally for mixed methods studies. In addition, we used a section of the MMAT for quantitative descriptive studies for a quantitative non-interventional cross-sectional study of telemedicine consultations [30]. Initial assessment was conducted by J.R., and L.T. double-checked the study quality and risk of bias.

### 2.7. Synthesis of Results

Given the heterogeneity of the studies in terms of study design, e-health approach, and outcomes, we decided not to perform a quantitative analysis of the studies because feasibility was limited, and utility was questionable. In contrast, based on recommendations for meaningful interpretation, we summarized the results in a qualitative synthesis of the reviewed studies [51,52].

## 3. Results

### 3.1. Study Characteristics

From the 27 included studies, we identified 21 unique e-health interventions from 8 countries (Brazil, Canada, Indonesia, Italy, New Zealand, Thailand, the United Kingdom, the United States). The majority, 25 studies, were interventions implemented by the research teams themselves; a total of 2 studies analyzed interventions not implemented by the researchers themselves and were classified as non-interventional studies [30,38]. A total of 10 studies addressed interventions for prevention and testing of HIV and other sexually transmitted infections (STIs), 7 studies addressed training and education for HCPs to acquire knowledge or reduce discrimination [14,15,16,58,59,60,61], 5 studies addressed mental health [46,62,63,64,65], 1 study addressed sexual health [66], 1 study addressed a telehealth project to promote trans health care outreach [44], 1 study addressed telemedicine and the impact of the COVID-19 pandemic on TGD health [30], 1 study addressed a massive open online course (MOOC) on LGBTQIA+ education and health services open to all user groups [67], and 1 study addressed online forums and social networks with health-related information [38]. In total, 14 studies were devoted to combined LGBTQIA+ samples or mixed samples with HCPs, 7 studies focused exclusively on HCPs, and 6 studies explicitly targeted only TGD people. In terms of age, 8 studies focused on young people under 25 [42,43,46,62,63,64,65,66], while the remaining 19 studies included people of any age. Most studies were characterized as pilot studies or approaches under evaluation given the constant innovation in the e-health field.

A variety of e-health approaches were included in the review. Among studies with TGD or combined LGBTQIA+ and mixed samples, there were seven studies of telehealth or telemedicine interventions (including five unique intervention approaches), six studies of mobile health apps (including five unique intervention approaches), three studies of game-based interventions (including two unique intervention approaches), three studies of online programs or courses, and one study of forums and online groups. The category of studies with HCPs only captures three studies on teleconsultations or e-consultations (as a joint unique intervention approach), and four studies on online training or courses. A summary of the included studies can be found in Table 2. Due to the volume of information, the tabulation of the synthesis of results is reported in the Supplementary Material. Tables S1 and S2 of Supplementary File S2 present an overview of the studies, divided into TGD or combined LGBTQIA+ and mixed studies (Table S1) and HCP studies (Table S2).

### 3.2. Risk of Bias in Individual Studies

A graphical and tabular representation of the critical appraisal using RoB 2, ROBINS-I, CASP, and MMAT can be found in Supplementary File S4.

#### 3.2.1. Quantitative Studies

Among the 17 quantitative studies included, there were 4 RCTs [42,43,63,70] and 13 non-randomized studies (including 4 comparative studies [65,68,69,72], 8 non-comparative single-arm studies [14,15,58,59,60,61,67,73], and 1 non-interventional study [30]). Across the five domains of RoB 2, the selected RCT studies were convincing. One game-based intervention scored in all domains with a low risk of bias [63]. However, the other three RCT studies also proved adequate overall for RoB 2, with some bias concerns purely related to possible baseline differences and slight non-adherence of individual participants. A total of 12 of the 13 non-randomized studies were assessed with the ROBINS-I tool in the seven domains with a moderate to serious overall risk of bias. For studies with a moderate overall risk of bias, this was due to participants being aware of their membership in the intervention group. The SPARX computerized cognitive behavioral self-help program suffered from serious performance bias due to a large attrition of participants across modules [65]. However, for the majority, it was the non-comparative studies that led to a serious overall risk of bias assessment due to a single-arm intervention via a possible attrition and detection bias. The lack of a comparison group made it impossible to assess the precise definition of the different treatment arms, leaving the intervention classification domain open with no information. A questionnaire study on telemedicine consultations during the COVID-19 pandemic only allowed an assessment with the MMAT due to its non-interventional study type [30]. This study covered the MMAT aspects. Weighing the critical appraisal, the heterogeneous quantitative studies allow sufficient representativeness to assess and discuss e-health approaches for TGD health.

#### 3.2.2. Qualitative Studies

Using the adapted CASP tool [56], the four included qualitative studies were found to follow the criteria. Three of the four studies were ostensibly (process) evaluation-style papers complementary to published project data and methodologically consistent with the CASP criteria [39,64,71]. The remaining qualitative study was a non-interventional study on forums and social networks for TGD people [38]. Overall, however, the design of all qualitative studies is plausible and provides an initial qualitative insight into e-health studies of TGD health.

#### 3.2.3. Mixed Methods Studies

According to the MMAT, the six included mixed methods studies [16,44,45,46,62,66] addressed the application aspects for mixed methods appropriately. The MMAT is a short instrument with five questions each to access the qualitative and quantitative parts of the study design and the quality of their integration. Measured against this, the researchers addressed their rationale adequately, combined the different methodological approaches appropriately, and integrated them comprehensibly in the presentation of results. All mixed methods studies had a pilot character, and five of them were non-randomized studies [16,44,45,62,66], each with different objectives and scopes. The evidence quality of the mixed methods studies should be evaluated cautiously and classified as preliminary results. The only RCT was an unpublished doctoral thesis on the effect of informative video tutorials on psychological well-being, which is also the only unpublished source in this review [46].

### 3.3. Synthesis of Results

#### 3.3.1. Content Focus and Health Approach of E-Health Services

Among the 20 included studies with TGD or combined LGBTQIA+ and mixed samples, half were devoted to e-health services for HIV/STI prevention. Various e-health approaches were used among these 10 studies with videoconference-based e-health services for assistance with HIV/STI testing and counseling, and app-based e-health services with text messages, quizzes, or interactive maps as various incentives to increase knowledge about HIV, testing options, or initiation of antiretroviral therapy. Four studies offered e-health-supported HIV/STI self-testing and online counseling on prevention and risk reduction (i.e., testing, condom use, PrEP, antiretroviral therapy) in the US TGD-specific RCT Project Moxie [42,43] with 202 participants (mean age 19 years) and in a Thai non-randomized unnamed study project [68,69] with 564 participants (largely MSM sample, including 17.55% trans women with a mean age of 28.1 years). Four other studies provided app-based content for knowledge gains related to HIV and PrEP and increased test readiness. This was true for the US MOTIVES [70,71], evaluated in a quasi-experimental randomized pilot study of 218 service users (largely MSM sample, including 30.2% and 36.7% trans women in the intervention groups with mean ages of 34.8 and 35.2 years, respectively), the Indonesian RUMAH SELA [72] non-randomized study project with 168 participants (largely MSM and drug user sample, of which 29.17% were trans women with a mean age of 25.6 years), and the US TGD-specific non-randomized usability study project Trans Women Connected [45] with 16 trans women and non-binary individuals (mean age 34.5 years). Two US studies focused on increasing the utilization of HIV-related health care, namely, weCare [39] (process evaluation with 32 participants, average age 25.2 years and including 27.78% trans women), and an unnamed eNavigation [73] addressed to 120 participants (largely MSM sample, including 14.17% trans women with an average age of 27.75 years).

Five studies focused on mental health. These include the Canadian non-randomized study project AFFIRM online, which tested cognitive behavioral therapy (CBT) online groups in depression, coping, stress appraisal, and hope with a sample of 96 LGBTQIA+ people (of whom 67.71% were TGD, with a mean age of 21.12 in the intervention group and 23.42 in the control group) [62]. In the US Singularities role play, a two-arm non-blinded pilot RCT, intervention participants played the superhuman Singular—a specially gifted character in a school overcoming challenges—and had access to a resource list for bullying, violence, and mental health, among other issues (sample of 240, of whom 47.08% were TGD with a mean age of 15.77) [63]. The fantasy world of SPARX shows the user game-based activities to reduce depression via seven CBT modules, which was tested in the LGBTQIA+ community in New Zealand with the general SPARX version [65] (891 finishers, including 1.57% TGD between 12 and 19 years of age) and an adapted LGBTQIA+ specific Rainbow SPARX version [64] in the United Kingdom (sample of 21 people, among the LGBTQIA+, 19.05% TGD with an average age of 17.9 years). The psychoeducational and trans-empowering UK program QueerViBE with six interactive YouTube video tutorials addressed psychological, social, and physical well-being (pilot RCT with 156 enrolled TGD people with an age range of 15 to 21 years) [46].

Another five studies had a separate thematic focus. The five-module US Queer Sex Ed program of a non-comparative pilot study provided sexual health education (sample of 202 LGBTQIA+ individuals, 6.93% of whom were TGD with a mean age of 17.91 years) [66]. An unnamed US e-health-supported intervention approach for 25 trans women of color (20% aged 18-25, with the remaining sample older or without age information) was designed to increase participants’ intention to seek trans health care in the future in a non-randomized pilot study of interactions with a peer health consultant [44]. A non-interventional Italian study examined how TGD people (sample of 108 aged 18 to 61, with a mean age of 34.3 years) used telemedicine consultations in terms of well-being and health-related quality of life and in the context of the COVID-19 pandemic [30]. An unnamed Brazilian MOOC open to all participants (sample of 582 completers, of whom 11.34% were TGD with a mean age of 29.4 years) educated about the rights, health, and social movements of the LGBTQIA+ community [67]. A non-interventional Italian study qualitatively analyzed health-related and community-related content of TGD-specific forums and Facebook groups and interviews of 16 selected actors of these networks (mean age 44 years, age range from 25 to 64 years) [38].

In addition to the 20 included community-focused studies, 7 studies examined HCPs only to bring them up to date on trans or LGBTQIA+ health care. Of these, three e-health studies in a US 3-year feasibility program were intensively dedicated to trans health care in the Veterans Health Administration (VHA) via video counseling and training approaches in terms of professional councils [14,15,16]. The webinar “Addressing the Needs for LGBTQ-Affirming Cancer Care: A Focus on Sexual and Gender Minority Prostate Cancer Survivors” focused on increasing the knowledge of 204 US HCPs [58]. The COLORS website training encouraged 44 US HCPs to engage in thought processes that would progressively change their LGBTQIA+-related knowledge, attitudes, and clinical practices [61]. The same was true for untitled videoconference-based interactive didactic sessions for 29 US residents and medical students [60]. An untitled LGBTQIA+ e-learning program for 307 Brazilian HCPs was designed to raise awareness of bias and discrimination in society and the health care system [59]. Overlapping features of these seven HCP-only sample studies are professional development and strengthening competence in dealing with persons of the TGD or LGBTQIA+ community.

#### 3.3.2. Execution of E-Health Services

Given the heterogeneity of e-health services, we observed a minimum intervention duration of less than an hour in the case of game-based interventions to a maximum intervention duration of several months in the case of complex programs. A range of mobile and non-mobile services were identified. In terms of e-health intervention delivery, 11 of the included studies analyzed services that offered videoconferencing. A variety of videoconferencing services were used, including Zoom [62], VSee [42,43], Digigone [44], a VHA in-house teleconsultation technology [14,15,16], service users’ preferred video platforms [68,69], or unspecified software [30,60]. For the videoconferencing services, service users had to have access to an internet-enabled device with a microphone and camera (e.g., laptop). While another 6 studies [39,45,70,71,72,73] dealt with mobile health apps, as mentioned above, for the remaining 10 included studies [38,46,58,59,61,63,64,65,66,67], web-accessible computers were in the foreground due to concepts such as games, educational programs, or forums.

Regarding individualization and interaction, the included e-health studies varied from none to diverse options. Individualization was present in videoconferencing-based services to the extent that a person’s requests were directly addressed. This allowed for differentiated interactions, even beyond the 1:1 setting (i.e., one-on-one meetings) in the case of HCP conferences. In this case, real or hypothetical cases could be discussed. The same is true for educational programs, when individuals were targeted for learning in didactically interactive elements. Individualization has not been possible in several e-health services without human interaction (i.e., services without videoconferencing services or forum components) because they are standardized. Individualization is given in game-based interventions in the form of customization of the game characters and the response of the game to individual gaming behavior [63,64,65]. The customization of these avatars is conditionally varied and rather limited in the games. A forced gender binary as in Rainbow SPARX when choosing a male or female avatar is criticized by LGBTQIA+ service users, also in the face of a possible gender-nonconforming customization of binary avatars (e.g., haircut, clothing), and they advocate for non-binary avatars [64].

Several e-health services relied on a range of offerings composed of different elements (i.e., complementing communication channels, supplementing an online support with an offline contact, expanding information content). In addition to video chats with a peer health consultant, one study with a videoconferencing service offered email, text, or telephone communication channels, which were preferred by participating trans women [44]. In a video-assisted HIV/STI prevention approach, when included in a mixed group, individuals (in contrast to an online-only or offline-only group) were subsequently offered offline HIV testing and counseling at a clinic after an online counseling session [68,69].

Information content in the included e-health studies was primarily text or video based. In some community-based educational programs, these services were supported by quizzes or tests to reinforce learning objectives [46,66,70,71,72]. The same applies to HCP-focused programs with questions and self-reflection tasks [58], module-based feedback [61], interactive role play sessions [60], or learning groups [59]. In VHA training for transgender care, HCPs were able to receive written feedback on their professional inquiries in e-consultations in addition to teleconsultations [15,16].

#### 3.3.3. Acceptability and Feasibility of E-Health Services

Overall acceptance and feasibility of e-health services were high in all included studies. In particular, e-health services with human interaction showed a high uptake. For example, these e-health interventions were experienced as helpful [62]. Facilitated, low-threshold access to a service and the accessibility of a professional were positively noted characteristics [44]. Study participants reported high satisfaction ratings [43]. In forums, the presence of and exchange with professionals were noted as positive, for example, when community management and specialist information are involved [38].

Mobile e-health services in the form of apps received high usability and acceptance scores across the studies. Service users valued the ease of integration into their busy lives [71]. In the app-based project weCare, service users praised a messaging function, whereby they could reach HCPs in an immediate way that would not be possible with real clinic visits [39].

The goals of professional development in HCP-only sample studies were met. HCPs rated themselves as more confident and competent to interact with TGD individuals after completing the program [14]. They noted an increase in competence regarding interpersonal skills in dealing with members of the LGBTQIA+ community and in knowledge of their specific needs, as well as new strategies and resources for their clinical work [58]. E-health interventions for HCPs were shown to be effective in reducing bias and identifying discrimination [59]. HCPs also cited a high willingness to recommend training opportunities to their colleagues [61].

Study participants recorded a strong continuity of participation in e-health services. Higher dropout rates were observed in isolated cases of game-based or course-based interventions [46,65,67]. Of 3000 MOOC course participants, only 582 completed the MOOC, which, with a completion rate of about 20% according to the authors, nevertheless exceeds the typical MOOC completion average of 2–10% [67]. In the video tutorial QueerViBE project, there was an attrition of 120 randomized people to 45 completed study participants [46]. In SPARX, of 9079 registered LGBTQIA+ youth, only 891 completed the SPARX modules to completion [65]. As a possible reason for this decline, the authors discussed that the appearance of SPARX was not inclusive enough due to the content and forced gender binary [65]. In addition, in the discussion for the Rainbow SPARX version, the game-based content was perceived by users as too simple and slow [64].

E-health services explicitly for TGD people consistently resulted in positive evaluations [30,43,44,45,46]. The same is true for e-health studies with combined community samples (i.e., LGBTQIA+ samples and mixed samples in the case of course formats designed to be open to all user groups) [38,39,62,63,64,65,66,67,69,70,71,72,73]. However, these studies provided only limited information on satisfaction and evaluation of the e-health content with regard to its suitability as TGD specific. Most studies were able to document statements regarding high uptake [72], but often those that used combined community samples did not analyze which modification of their e-health intervention could better meet the specific needs of TGD people. There is evidence of this when study participants in e-health services for the entire LGBTQIA+ community were given the opportunity to critique. Referring to the Queer Sex Ed program, one person criticized the program in response to the question *“What did you dislike about this program?”*: “I disliked the very limited amount of trans* information in this program. Sexual health for trans* people is something that I can’t seem to find anywhere.” [66] (p. 227).

#### 3.3.4. Effectiveness of E-Health Services

In terms of outcome measures, the different e-health studies proved to be very heterogeneous (see Supplementary File S2). Most studies tended to have specific outcomes to assess the feasibility and acceptability of their specific e-health services or the effectiveness of these in terms of improving health or extending health services [15,66,68,71]. Overall, there was no evidence in the included studies that an e-health service would be negative or harmful to health or quality of life [14,16,43,44,45,58,61,62,63,64,66,71]. E-health services tended to be more effective in positively affecting study-specific outcomes [30,43,44,45,46,62,63,66,67,69,70,72].

In summary, the 10 included e-health studies on HIV/STI prevention succeeded in contributing to increased knowledge, increased willingness to test, and uptake of treatment options. In one videoconference-based e-health project, compared with baseline data, knowledge about HIV and PrEP increased over time, as did willingness to use PrEP, and after 3 months, there was generally a higher testing frequency in the short term, although this declined again in the medium term after 6 months [42,43]. Satisfaction with the e-health service was very high, as counselors were described as very knowledgeable, and participants would be happy to use the program again and recommend it to others [43]. A similar videoconference-based e-health service offering with an online-only, offline-only, and mixed blended care approach proved effective, such that more individuals learned their HIV status, but at the same time, if the HIV status was positive, only 52.8% in the online group started antiretroviral therapy directly compared with the offline (84.8%) and mixed groups (77.8%) [68,69]. As a result of app-based e-health services, knowledge of HIV and PrEP increased [45,70,72], high acceptability and feasibility of the service due to low time commitments were reported [71], condomless sex decreased [72], study participants tested themselves more frequently after an intervention [72], and viral suppression was observed more frequently after intervention [73]. High levels of satisfaction were identified with in-app games and interactive maps [72], usability [45], and more community-oriented communication with HCPs [39]. In addition, self-efficacy in one’s ability to find LGBTQIA+-friendly services increased [45].

The five included mental health-focused e-health studies appeared to be able to contribute to quality of life improvements. After group therapy in AFFIRM Online, depression scores decreased, coping strategies increased, and knowledge of LGBTQIA+- and mental health-related issues increased in the intervention group [62]. The video tutorial-based QueerViBE e-health program contributed to TGD empowerment and increases in psychological, social, and physical well-being [46]. After completing the Singularities game, study participants used online resources more frequently and experienced less cyberbullying and victimization, and the incidence of binge drinking decreased [63]. The CBT-modularized game SPARX or Rainbow SPARX was experienced by the LGBTQIA+ community as a useful tool, although only cautiously recommended to others [64,65]. Up-to-dateness and TGD-specific game content are important to users; thus, SPARX, as a trans-non-specific game, did not lead to a decrease in depression among TGD people, while it did among cis members of the LGBTQIA+ community [64,65].

The five remaining e-health studies with community-based samples focused specifically on improving health or quality of life through the specific service in their outcomes. The evaluation of the Queer Sex Ed program showed overwhelmingly high satisfaction and learning gains in sexual health issues, with slight disapproval for little TGD-specific information [66]. Telehealth-supported interventions with a peer health consultant increased trans women of color’s likelihood of seeking trans health care help in the future, their resistance and skepticism were overcome in supportive interactions with their peer health consultant, and they were more open to receiving further health care services [44]. In a quantitative study, the availability of telehealth consultations during the COVID-19 pandemic was associated with a lower impact on COVID-19-related, general, and TGD-specific mental health through the event [30]. A MOOC revealed to community and noncommunity members that they previously knew little about social movements, NGOs, and LGBTQIA+ health services, and the MOOC led to an increase in knowledge [67]. In forums, TGD users shared helpful links to HCPs and LGBTQIA+ organizations for medical inquiries; in addition, the presence of HCPs in forums for professional questions was seen as positive [38].

Finally, the last seven studies of e-health services with HCPs largely met their goals of training and education. After completing the teleconsultation- and e-consultation-based VHA services, HCPs reported more confidence and rated the didactics and consultations as helpful [14,15,16]. Predominantly, they used the VHA program for questions about hormone therapy, primary care, and mental health, and to discuss treatment plans, treatment recommendations, and information needs [14,15,16]. Several individual online training programs and courses were positively received by HCPs. They promoted knowledge growth and clinical expertise on LGBTQIA+ [58,60,61], specifically including affirming and culturally sensitive strategies and specific needs in prostate cancer treatment [58]. They also contributed to the reduction in prejudice and more awareness of discrimination in the health care system [59].

## 4. Discussion

### 4.1. Summary of Findings

According to social-ecological models, TGD people are significantly at risk for impaired general or mental health influenced by multiple stigma [3,9]. The literature discusses whether and how this situation could be improved through e-health services tailored for TGD people and facilitated contacts with specialized HCPs (e.g., access and counseling for gender-affirming medical interventions) [11]. The current systematic review intended to identify and evaluate e-health approaches, in terms of (1) the scope of TGD health in the evaluated e-health services, (2) the effectiveness of various e-health services in terms of improving study-specific indicators for better trans health care, and (3) overall acceptance and feasibility for trans health care.

#### 4.1.1. Assessment of the First Research Question: Which E-Health Services Have Been Empirically Tested That Improve the Health of TGD People Directly through Treatments for TGD People or Indirectly through Qualifying HCPs?

Related to the first research question, with 27 included studies from 8 different countries, including 21 unique e-health approaches, we were able to identify projects for each of the e-health sub-concepts presented in the introduction. Measured against the fact that the oldest included study was from 2015, and 19 studies were published in 2019 or later, there has been clear innovation for TGD people in recent years with many pilot projects. A total of 14 studies analyzed e-health services for the entire LGBTQIA+ community, 7 studies analyzed e-health services for HCPs only, and 6 studies analyzed e-health services for TGD people only. This observation is in line with our expectation that there is a lack of research and e-health projects exclusively for TGD people. In response to the first research question, the development and research of e-health interventions for TGD are still in their infancy, but there has recently been a growing trend of pilot e-health projects for the TGD and LGBTQIA+ community. Still, there is a need to provide tailored interventions to subpopulations such as TGD people, as they have specific health-related needs [47]. The health care needs of TGD people require special attention, as they are often insufficiently recognized in the health care system, among HCPs, and even within the LGBTQIA+ community [74]. This is reflected in the dislike of programs that do not provide information specialized for TGD [66] or binary characters with which non-binary people cannot identify [64]. This may also have contributed to the lack of improvement in depression among TGD individuals in the SPARX program [65]. Exclusion of TGD people, lack of knowledge of their needs, assumptions of a universal transgender experience, and denials of transphobia are among the many forms of microaggressions that TGD people experience in contact with HCPs and LGBTQIA+ people [74]. Therefore, non-discriminatory, TGD-sensitive training must be provided to HCPs to improve care [9,75], as highlighted by the reviewed research on HCP training and education [14,15,16,58,59,60,61]. Otherwise, invisibility of TGD people can result in emotional responses to (micro)aggression such as anger, betrayal, hopelessness, exhaustion, and feelings of being invalidated and misunderstood, along with lower service utilization as a result of experiences of discrimination and mistreatment, perpetuating barriers to trans health care [4,5,6,74]. Utilization of trans health care seems more likely with confidence in the self and others after participation in TGD-specific e-health services, which enables further connection to TGD-related health services (e.g., HIV prevention, gender-affirming therapy) [44]. Particularly in this case, affirming and empowering e-health services for the TGD community, such as the approaches included in the current systematic review, are important for appropriate health care, self-acceptance, overcoming stigma, and improving mental health [46,62].

#### 4.1.2. Assessment of the Second Research Question: How Effective Are E-Health Services in Improving the Health of TGD People?

Regarding the second research question of effectiveness, the results across studies indicated that participation in e-health services was beneficial for the service users and that they were able to achieve improvements in outcomes concerning health or quality of life [38,42,43,44,45,46,62,63,66,67,70,72,73]. Surprisingly, the majority of the e-health studies presented did not include variables such as rural residence, education, and isolation in their data collection and analyses. Therefore, it is not possible at this point to answer the extent to which hard-to-reach TGD populations benefit from e-health services with respect to these variables. Few studies documented place of residence and education [15,16,59,66,67,69]. The Queer Sex Program [66] mentioned place of residence only descriptively (urban 85.3%, rural 12.6%) without deeper discussion of it, as did the Brazilian MOOC [67] (urban 88.8%, rural 6.2%). In the Thai HIV e-health project, where service users were free to choose one of three intervention groups, education level, income, and HIV prevention knowledge were significantly higher among the blended care mixed group compared to the online-only and offline-only groups [69]. In the Brazilian e-learning program for HCPs, pre-intervention prejudice levels were higher among individuals with lower levels of education and religious beliefs, and those residing in smaller cities compared to individuals from areas with a higher population, but, at the same time, they were able to reduce prejudice more in percentage terms after the intervention due to the increase in knowledge [59]. The VHA program discussed that rural HCPs in particular might benefit from consultation services because they otherwise lacked contact with more experienced colleagues in their immediate area [15], but it was unable to find differences between urban and rural HCPs in use or non-use of consultation services in post hoc analyses [16].

By feedback of service users, no disadvantages were derived for the presented e-health services (e.g., factors that could be negative or harmful for health). As a possible disadvantage for TGD people, it would be worth considering whether they can be sufficiently addressed in e-health services for combined community samples. The included studies of this type did not provide information on whether LGBTQIA+ projects are sufficiently adequate for TGD individuals. In the case of HIV/STI care e-health services, it should at least be noted for combined community samples that, in addition to the dominant group of MSM, only trans women were included as representatives of the TGD community and as a particularly vulnerable group at high risk for HIV [39,68,69,70,71,72,73]. For the TGD community as a whole, from an equity perspective, trans men and gender diverse individuals should also be included [76]. In TGD-specific HIV/STI care e-health services, specific tailoring and inclusion of multiple TGD individuals were possible, meaning that the disadvantages that otherwise exist in reality due to the greater attention paid to trans women in HIV prevention services compared to trans men or gender diverse individuals were not given [42,43]. At the same time, as in the seven included HCP-focused studies, TGD people need HCPs to improve their professional expertise on health care for the TGD and LGBTQIA+ community to prevent mistreatment in health care settings [9,74]. From this perspective, the effectiveness and benefits of various e-health services for different target groups could include reducing TGD stigma structurally (i.e., by expanding gender-affirming health offerings to digital e-health components), interpersonally (i.e., by knowing about supportive interactions within an e-health intervention), and individually (i.e., through self-acceptance and active seeking out of health offerings) [9,11,13].

#### 4.1.3. Assessment of the Third Research Question: How Acceptable and Feasible Are E-Health Services for Trans Health Care?

The third research question dealt with the overall usefulness of e-health services for TGD people, whether they are considered acceptable and feasible. Strictly speaking, there were no comparable assessments of e-health services in terms of acceptability and feasibility between the studies, but service user feedback was specific to the particular intervention approach, meaning conclusions could be drawn in this regard. The integration of e-health services into people’s everyday lives, which is flexible and low cost and not tied to a specific location, should be emphasized [71]. This allows HCPs to contact service users directly in their familiar environment in a way that would not be possible without e-health services [39]. TGD as well as LGBTQIA+ individuals could benefit in the long run if HCPs are trained through e-health services. A significant issue with e-health services for TGD service users is whether a treatment service should be classified as too basic [64,66]. If TGD service users classify content as too generic and not optimally inclusive due to an enforced gender binary, as in a game-based intervention, this tends to be counterproductive to the commitment to participate [64]. Further to the included studies, according to a recent qualitative study (this study was captured via the literature search but excluded due to its non-interventional study type and general discussion of generic TGD-non-specific gamified interventions) with 14 TGD adolescents between 11 and 18 years, focus group results indicated that potential users of game-based e-health interventions value gender inclusivity, up-to-dateness, and personalization of content and prefer a focus on prevention and mental health [77]. At the same time, this study supported several previously mentioned mental health-influencing stigma sources such as, on the stressful side, transphobia, lack of support opportunities, lack of gender representation, and negative encounters online, and, on the health-promoting side, helpful social media and online information, education about LGBTQIA+, and finding likeminded peers [77]. In the case of games, addressing these issues and adapting future e-health interventions for TGD could potentially help build resilience [77].

In the case of HIV/STI e-health services, care should be taken to ensure that online concepts in the sense of blended care are meaningfully interlinked with face-to-face contacts with HCPs. Given the included e-health studies, it seems to be more helpful if service users obtain information and advice online and are subsequently referred to testing centers and HIV specialists [43,45,70,71,72]. Thus, in a videoconference-based e-health service offering with an online-only, offline-only, and mixed blended care approach, antiretroviral therapy was started directly less frequently in the online-only group for HIV-positive status in terms of relative frequency [69]. At the same time, the online-only intervention group provided the opportunity to support individuals with HIV testing who would not otherwise have their HIV status tested without this online support [69]. In highly specialized HIV care, a blended care approach might be the more feasible approach for e-health services compared to an online-only approach.

Other profound benefits for TGD people from an e-health service will probably occur when TGD people can engage with HCPs, such as a peer health consultant, who understands them and their needs with a personal background of experience and helps them understand their situation and needs [44]. Such HCPs can provide initial support, especially when dealing with cases of high stigma where trust in oneself and others must first be built. This can reinforce a TGD person to seek trans health care and helps reduce barriers to access. Many TGD people are very active in using online forums and communities, where they often experience validation and appreciation for the first time, as well as helpful tips [1,2]. In an appropriate digital medium, HCPs could reach out to TGD people directly in a low-threshold way to help them access trans health care [38].

### 4.2. Implications and Future Directions

Overall, the results of the current systematic review indicate that adding e-health services to the range of treatments for TGD people offers promising solutions for improving health and quality of life. Thus, it is advisable to go beyond the format of offline care with e-health services. Evidence on the effectiveness of e-health services addressing aspects of trans health care appears to be consistent with findings on e-health services in other application areas such as depression, anxiety, alcohol prevention, or stress management [22,23,24,25,26,27]. In RCTs, systematic reviews, and meta-analyses on these application areas, e-health services are shown to be feasible, acceptable, and effective in reducing symptoms and promoting health and are discussed in publications as helpful complements to offline care [22,23,24,25,26,27]. In the future, it would be a reasonable consideration for HCPs to approach TGD people in their immediate life reality for a more barrier-free offer, so that, through an e-health service, the setting of a hospital or doctor’s office associated with barriers is not initially present. Reflection processes about the value of taking care of one’s own health appear to be able to be triggered in a familiar private setting of a service user by e-health services, so that they minimize stigma and TGD people may seek out offline offers of trans health care for the first time or to a greater extent than before. In view of this, it seems recommendable to integrate e-health services into existing treatment concepts (e.g., HIV/STI prevention, sessions with a mental health professional).

Several of the included studies had multimedia elements in their e-health services, such as video calls, text messaging, games, or informational videos, so a generalizing statement about which element is best or most likely to improve trans health care cannot be made. E-health approaches of various types are likely to decrease barriers to health care by overcoming distance and time flexibility. Research suggests that trans-sensitive e-health services are likely to help service users recognize discrimination, build coping strategies, and promote self-acceptance [46,62]. For the future, it is suggested that training and education opportunities for HCPs continue to advance so that discrimination in the health care system decreases. Special attention should be paid to the interdisciplinary character of trans health care [78,79]. For this reason, e-health services for HCPs should provide discipline-specific training that enables HCPs to train in their discipline, such as endocrinology, according to current TGD health guidelines [80,81,82,83,84]. In addition to traditional face-to-face training, a variety of e-health training opportunities are emerging, ranging from on-demand courses that reach large numbers of HCPs (e.g., MOOCs [67]) to customized specialty councils for small group work [14,15,16]. Recent findings reveal the desire of HCPs for training to improve their competencies and skills and provide evidence for developing e-health services for HCPs [85]. Interventional studies of e-health services that have actually been implemented and evaluated [42,43,44,45], as well as observational studies that did not implement an intervention themselves but evaluated an e-health offering applied in practice [30,38], provide a starting point for further research. As with all types of interventions, e-health, with its various sub-concepts, requires in-depth research on the effectiveness, acceptability, and feasibility of the treatment in question. Therefore, we see the need for researchers to consider, develop, and evaluate e-health services, including for TGD people [86,87,88]. This would also address the research obstacle of the lack of specific data for the TGD subpopulation of the LGBTQIA+ community [89,90].

Given the sparse evidence, future studies should evaluate the issue of differential indication. More in-depth analyses are needed to assess for whom TGD-specific e-health services are appropriate. For severely burdened TGD people, as in the case of suicidality, it would be necessary to assess at what point they could benefit from e-health services and when a contraindication exists. In case of a contraindication in crises and the risk of endangering oneself or others, we recommend, from a clinical point of view, the implementation of a warning box in an e-health service that refers to emergency numbers and (psychiatric) emergency rooms. Additionally, resources can be listed for immediate help. A resource list should include aspects of emergency contacts, direct contacts, and specialized local services. An interactive map in mobile apps, in which service users can locate nearby specialized agencies, proves to be practical [45,72].

Different elements such as individual or group settings, videoconferencing services, phone calls, messaging, course modules, informational videos, games, quizzes, forums, resource sharing, and referrals to specialized HCPs can be considered for the development of multimedia e-health programs. From an equity perspective, sign language, easy-to-read language, and multilingualism should be considered in the design. These aspects in particular are rarely, if ever, mentioned in the literature but are significant for respectful inclusion of all people [39,91,92]. Finally, for e-health service developments, we recommend that content be updated regularly to stay current. Otherwise, content quickly becomes outdated, and users become bored or feel unaddressed [64,66].

### 4.3. Strengths and Limitations

The main strength of our systematic review is that, to our knowledge, it is the first of its kind to assess the current state of research on various e-health sub-concepts that could improve trans health care. Telemedicine is gradually gaining interest following similar reviews [50], but our systematic review is the first to provide a comprehensive overview of e-health with various sub-concepts and their effectiveness, acceptability, and feasibility when used in trans health care. Implementation of e-health in trans health care is new, and although this field is growing, evaluation research has been sparse. Therefore, we included studies of different designs with different outcome measures. Based on the available evidence, a detailed quantitative analysis of the included studies proved to be impractical and not meaningful in terms of content, so we compared studies in a descriptive manner in a qualitative content synthesis. Since we limited the start of our search to the year 2000, we could not exclude having overlooked earlier studies. However, we consider the probability to be low, as the oldest included study was published in 2015 [66]. Furthermore, with the inclusion criterion of interventional or observational studies, a priori we excluded studies that simply mentioned presumed benefits of e-health services and did not evaluate interventions for TGD health. However, because of a lack of intervention, these studies did not allow us to draw conclusions about the focus of the systematic review on the effectiveness, acceptability, and feasibility of an e-health service. While it is critical to note that only 6 studies evaluated e-health services exclusively for TGD people (plus a few e-health services for HCPs on TGD health) and the inclusive search strategy may have been designed too broadly, we believe that the 27 included e-health studies reflect reality to a certain extent. TGD people are more likely to be included primarily in services for the LGBTQIA+ community or secondarily when HCPs educate themselves about TGD or LGBTQIA+ health care needs. In this respect, the intersection of service user groups in various e-health services reflects reality and enables digital care to be set up for TGD people: (1) by TGD people being targeted in TGD-specific or LGBTQIA+-inclusive e-health services; (2) by supporting barrier and stigma reduction through facilitating online access to health care services and expanding training opportunities for HCPs.

Inclusion of e-health studies for the treatment of diverse populations and continued training of HCPs will likely need to be addressed simultaneously in future systematic reviews until specialized trans health care and offerings for gender-affirming treatment are established in the e-health field worldwide [82]. Only after further research on e-health can services for TGD people and HCPs be evaluated in depth separately; clearly, inclusive consideration of diverse populations and ongoing education of HCPs should continue after e-health has been implemented into the care structure. In our systematic review, many of the included studies from different countries included TGD people as part of the LGBTQIA+ community in their respective e-health service. That being said, other systematic reviews also demonstrate the general challenge of adequately representing one or more subgroups of the LGBTQIA+ community, as there are not many studies in each case [47]. Although not all of the studies included in our systematic review focus exclusively on TGD people, they do provide preliminary practical lessons for implementing e-health services in trans health care (e.g., barrier-free enrollment in a service, diversity-competent HCPs, gender inclusivity). We conducted this systematic review during the global COVID-19 pandemic, and therefore an increase in e-health services for TGD people was expected due to social distancing. Specifically, in the pandemic, TGD people are heavily burdened by mental health deterioration, restrictions, and limited access to medical care and low-threshold services (e.g., TGD support groups) and could benefit from e-health services [93,94]. In consequence, we anticipate that more e-health studies will follow in this regard in the near future [95,96]. Additionally, given excluded study protocols in this review for which published data were not available, updating the assessments of our systematic review is recommended in future papers. Finally, we note that the majority of the 27 included studies are not RCTs and have methodological problems related to risk of bias (see Supplementary File S4). Based on our systematic review, study type (e.g., RCTs) and methodological quality (e.g., appropriate quantitative and qualitative data collection) are considered critical variables for future research. Developers and researchers should systematically evaluate their e-health services (e.g., in terms of context, implementation, and mechanisms of impact) [97]. With this in mind, we are currently conducting our own RCT on the e-health service i^2^TransHealth [98].

## 5. Conclusions

Considering the stigmatization of TGD people, their health needs, and the need to reduce access barriers to trans health care [9], this systematic review focused on e-health services as potential solutions to the known problems. Findings from pioneering work in recent years indicate that e-health services are able to improve trans health care through an expansion of offerings. This is made possible through e-health projects exclusively for TGD people, and for the LGBTQIA+ community in general, and continuing education for HCPs. However, TGD people still prove to be an underrepresented group in e-health research, which is why the need for inclusive consideration of TGD people stands out strongly. The lack of studies should not lead to TGD people being further lost sight of in e-health research. Rather, we encourage researchers to launch their own e-health pilot projects to improve trans health care. From our perspective, based on this review and ethical and clinical considerations, we recommend e-health services pay attention to tailored, TGD-specific design and regular updating of their content [13]. When implemented appropriately, e-health services might provide a safe, secure setting in which service users can receive confidential, low-cost support remotely that is compatible with their personal schedules. In this context, e-health services do not replace traditional standard care but can usefully complement it [13]. In this way, TGD people can be targeted who would not otherwise have trust in trans health care or who do not seek it out for other reasons. Especially for remotely living TGD people from rural or suburban areas, research discusses a benefit of e-health to overcome long distances via the digital medium, which needs further empirical verification [11,13]. E-health services for the TGD or LGBTQIA+ community can contribute to gender equality if they welcome persons of all identities and expressions [75]. This opens the door for intersectional perspectives and the reduction in stigma through marginalization regardless of gender identity or sexual orientation.

## Figures and Tables

**Table 1:** E-health sub-concepts and their fields of application.

Sub-Concept	Brief Description of the Fields of Application
Programs	Self-contained systems with content for autodidactic learning or improving health or well-being, e.g., modules, informational texts, videos, games.
Telehealth	Video consultations or counseling by HCPs, presentation of preventive measures for health and well-being.
Telemedicine	Video consultations by medical HCPs, distance overcoming medical exams and advice, medical focus on health.
mHealth	Mobile apps using mobile technologies with or without guidance from HCPs.
Blended care	Combination of internet interventions via video consultation and regular face-to-face treatment.
Social media health	Animating users to adopt health-promoting behaviors through social media.
Further approaches	Self-help, coaching, chats, forums, push notifications, and appointment reminders.

**Table 2:** Summary of e-health services on transgender and gender diverse health concerns.

Intervention Name	Outcomes	Author(s)	Year	Location	Study Design	Sample, *n*, Mean Age (SD)	E	A	F	Effects
Telehealth or Telemedicine Interventions										
AFFIRM Online	Depression (D), Coping (C), Stress Appraisal (SA), Hope (H)	Craig et al. [62]	2021	Canada	MM: non-randomized	Mixed sample with TGD, *n* = 96, intervention: 21.17 (4.52), control: 23.42 (3.41)	x	x	x	D ↓ C ↑ SA ↑ H –
Project Moxie	HIV and STI testing	Sharma et al. [42]	2019	USA	QNT: pilot RCT	TGD only, *n* = 186, age groups: 15–18, 19–24, mean age: 19	n/a	n/a	n/a	n/a
	HIV and STI testing	Stephenson et al. [43]	2020	USA	QNT: pilot RCT	TGD only, *n* = 202, age groups: 15–17, 18–20, 21–24	x	x	x	overall ↑
Telemedicine consultations in Italy	Impact of Event (IES), Depression (D), Health-related Quality of Life (QoL)	Gava et al. [30]	2021	Italy	QNT: NIS	TGD only, *n* = 108, 34.3 (11.7)	x	x	n/a	IES ↓ D – QoL –
Unnamed online HIV counselling and testing	key factors for choosing service options	Phanuphak et al. [68]	2018	Thailand	QNT: non-randomized	Mixed sample with TGD, *n* = 564, 27.9 (7.2)	n/a	x	x	n/a
	linkages to HIV confirmatory testing and ART initiation (AI)	Phanuphak et al. [69]	2020	Thailand	QNT: non-randomized	see above	n/a	x	x	HIV testing ↑, AI challenges in online group
Unnamed telehealth intervention	intention to seek care, receipt of care	Magnus et al. [44]	2018	USA	MM: non-randomized pilot study	TGD only, *n* = 25, age groups: 18–25, >25	x	x	x	overall ↑
Mobile health apps										
MOTIVES	HIV knowledge and frequency of HIV testing	MacCarthy et al. [70]	2020	USA	QNT: quasi-experimental randomized pilot study	Mixed sample with TGD, *n* = 218, Information Only: 34.8, Information Plus (IP): 35.2, Comparisons: 33.7	x	n/a	n/a	testing ↑, knowledge ↑ only in IP group
	process evaluation	MacCarthy et al. [71]	2021	USA	QUAL: quasi-experimental randomized pilot study	Mixed sample with TGD, *n* = 41, 37.36	x	x	x	positive assessments
RUMAH SELA	HIV prevention knowledge	Garg et al. [72]	2020	Indonesia	QNT: non-randomized prospective intervention cohort study	Mixed sample with TGD, *n* = 168, trans women: 25.6 (3.0)	x	x	x	overall ↑
Trans Women Connected	usability, PrEP knowledge, self-efficacy, social support	Sun et al. [45]	2020	USA	MM: non-randomized study	TGD only, *n* = 16, 34.5 (9.28)	n/a	x	n/a	overall ↑
Unnamed eNavigation	HIV care continuum outcomes	Arayasirikul et al. [73]	2020	USA	QNT: non-randomized non-comparative	Mixed sample with TGD, *n* = 120, 27.75 (4.07)	x	n/a	n/a	undetectable viral load ↑
weCare	process evaluation	Tanner et al. [39]	2020	USA	QUAL: non-randomized non-comparative	Mixed sample with TGD, *n* = 32, 25.2 (3.79)	n/a	n/a	n/a	positive assessments
Game-based interventions										
Singularities	several health outcomes	Egan et al. [63]	2021	USA	QNT: 2-arm non-blinded pilot RCT	Mixed sample with TGD, *n* = 240, 15.77	x	x	x	victimization ↓ binge alcohol use ↓ marijuana use ↓
Rainbow SPARX/ SPARX	use of internet for mental health	Lucassen et al. [64]	2018	United Kingdom	QUAL: non-randomized non-comparative	Mixed sample with TGD, *n* = 21 youth and 6 HCPs, 17.9	x	x	n/a	assessments for necessary updating and refinement
	depressive symptoms	Lucassen et al. [65]	2020	New Zealand	QNT: non-randomized	Mixed sample with TGD, *n* = 891 finishers, age groups: 12–15, 16–19	x	n/a	n/a	↓ in cis people, – in TGD
Online programs or courses										
Queer Sex Ed	sexual health outcomes	Mustanski et al. [66]	2015	USA	MM: non-randomized non-comparative pilot study	Mixed sample with TGD, *n* = 202, 17.91	x	x	x	overall ↑
Queer ViBE	psychological and physical well-being	Martin [46]	2019	United Kingdom	MM: pilot RCT	TGD only, *n* = 120 randomized, intervention: 18.09 (1.70), control: 17.73 (1.64)	x	x	x	overall ↑
Unnamed MOOC	completion rate, study participants characteristics	Canavese et al. [67]	2020	Brazil	QNT: non-randomized non-comparative pilot study	Mixed sample with TGD, *n* = 582 completers, 29.4 (9.9)	x	n/a	n/a	learning about LGBTI+ topics ↑
Forums and online groups										
Internet: forums and social networks	reasons for online interactions, types of support	Cipolletta et al. [38]	2017	Italy	QUAL: NIS	Mixed sample with TGD, *n* = 16 interviewees, 44 (range: 25–64 years)	n/a	n/a	n/a	benefit through help and support
Teleconsultations or e-consultations										
VHA Trans-gender SCAN-ECHO/VHA e-consultation	confidence in providing care to trans veterans	Kauth et al. [14]	2015	USA	QNT: non-comparative pilot study	HCPs only, *n* = 33, age: n/a	x	n/a	x	overall ↑
	typical questions from providers	Shipherd et al. [15]	2016	USA	QNT: non-comparative pilot study	HCPs only, *n* = 303 e-consults, age: n/a	n/a	n/a	x	indications for feasibility due to high usage
	providers’ program experiences, methods for improving program use	Blosnich et al. [16]	2019	USA	MM: non-randomized study	HCPs only, *n* = 15 interviewees, 53 survey participants, age: n/a	n/a	n/a	n/a	high reported usefulness
Online trainings or courses										
LGBTQ-Affirming Cancer Care	knowledge increase, satisfaction with training	Pratt-Chapman et al. [58]	2020	USA	QNT: non-randomized non-comparative	HCPs only, *n* = 204, age groups from 21–29 to 60 or older	x	x	n/a	overall ↑
COLORS training	LGBT-related knowledge, attitudes, clinical practices	Seay et al. [61]	2020	USA	QNT: non-randomized non-comparative pilot study	HCPs only, *n* = 44, 47.4 (9.3)	x	x	x	overall ↑
Unnamed interactive on-line didactic session	clinical skills, clinical preparedness, knowledge on LGBTQ health	Barrett et al. [60]	2021	USA	QNT: non-randomized non-comparative	HCPs only, *n* = 29, 29 (5.0)	x	n/a	n/a	overall ↑
Unnamed web-based intervention	prejudices, prevalence of discrimination	Costa et al. [59]	2016	Brazil	QNT: non-randomized non-comparative	HCPs only, *n* = 307, 34.52 (9.40)	x	n/a	n/a	overall ↓

All included studies allow statements on effectiveness, acceptability, and feasibility. In this respect, the x only indicates whether a study considered specific measurement instruments for these three aspects in the study design. Abbreviations and symbols: - no change, ↑ significant increase, ↓ significant decrease, A = acceptability, AI = antiretroviral therapy initiation, C = coping, D = depression, E = effectiveness, F = feasibility, H = hope, IES = impact of event, IP = information plus group (intervention group), MM = mixed methods study, MOOC = massive open online course, NIS = non-interventional study, QNT = quantitative study, QoL = health-related quality of life, QUAL = qualitative study, SA = stress appraisal, VHA = Veterans Health Administration.

## Data Availability

Data are included in the article and Supplementary Materials.

## Supplementary Materials

The following supporting information can be downloaded at https://www.mdpi.com/article/10.3390/jcm11041090/s1: Supplementary File S1: Figure S1. Flow diagram showing the selection process for the systematic review [99]. Supplementary File S2: Table S1. Systematic review summary of included studies with TGD or combined LGBTQIA+ and mixed samples, Table S2. Systematic review summary of included studies with health care professional-only samples. Supplementary File S3: Keywords and search strategies used for the systematic review. Supplementary File S4: Figure S2. Results of critical appraisal of RCTs according to RoB 2, Figure S3. Results of critical appraisal of non-randomized quantitative studies according to ROBINS-I, Table S3. Results of critical appraisal of qualitative studies, Table S4. Results of critical appraisal of mixed methods and remaining studies. Supplementary File S5: PRISMA 2020 Checklist.
