# Supplementary material for: Need for Inclusive Consideration of Transgender and Gender Diverse People in E-Health Services: A Systematic Review
Source: J Clin Med. 2022 Feb 18;11(4):1090. doi: 10.3390/jcm11041090 (PMC8880545; doi:10.3390/jcm11041090)
Supplement: Supplementary file 1 [file jcm-11-01090-s001.zip › Supplementary File S1_PRISMA_2020_flow_diagram_new_SRs_v2_Review_Trans.pdf]

Supplementary File S1: PRISMA 2020 flow diagram for systematic review

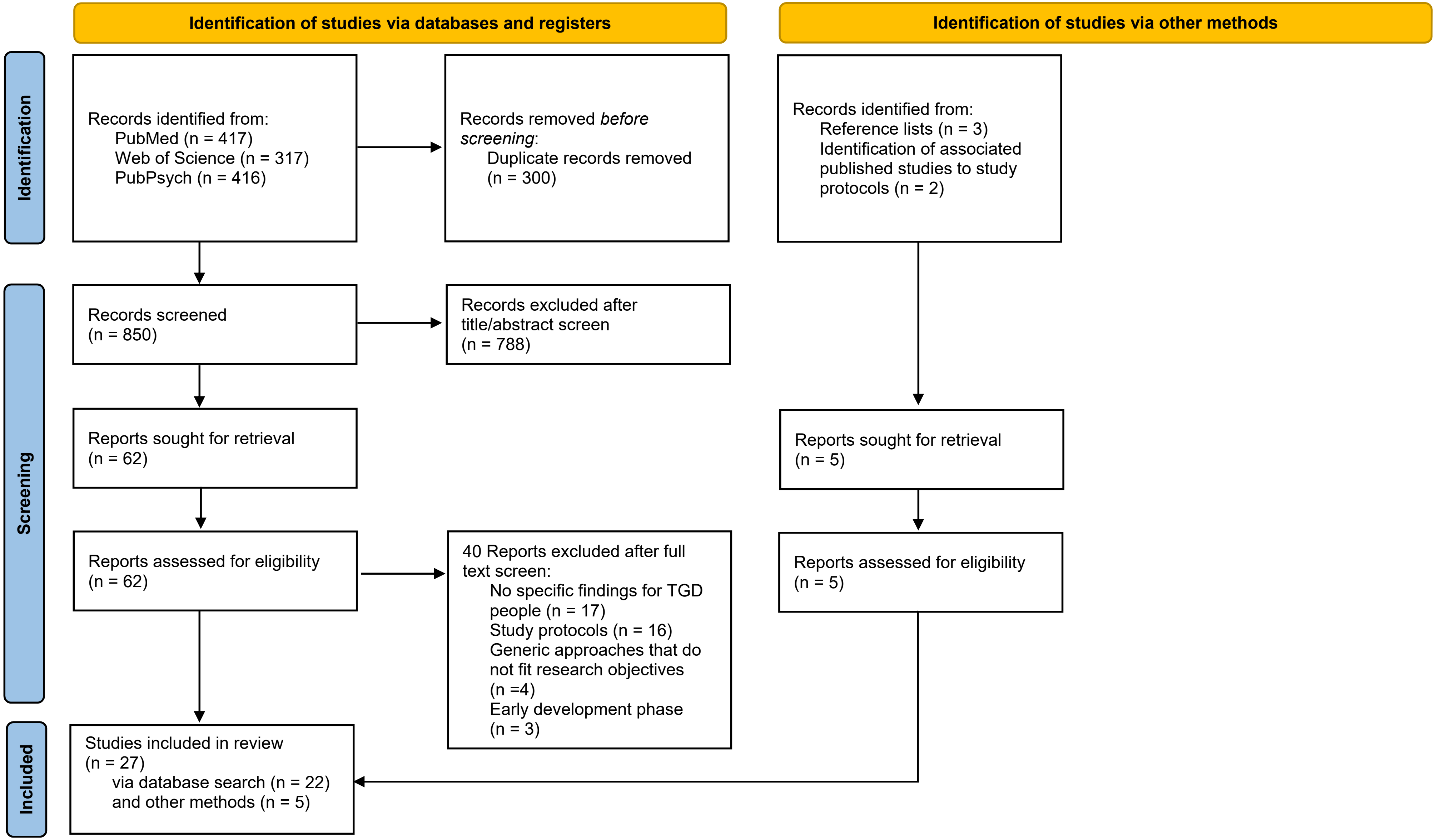

Figure S1. Flow diagram showing the selection process for the systematic review.

From: Page MJ, McKenzie JE, Bossuyt PM, Boutron I, Hoffmann TC, Mulrow CD, et al. The PRISMA 2020 statement: an updated guideline for reporting systematic reviews. BMJ 2021;372:n71. doi: 10.1136/bmj.n71. For more information, visit: http://www.prisma-statement.org/
