# Supplementary material for: Need for Inclusive Consideration of Transgender and Gender Diverse People in E-Health Services: A Systematic Review
Source: J Clin Med. 2022 Feb 18;11(4):1090. doi: 10.3390/jcm11041090 (PMC8880545; doi:10.3390/jcm11041090)
Supplement: Supplementary file 1 [file jcm-11-01090-s001.zip › Supplementary File S2_Tables_Systematic_Review_Findings.pdf]

Supplementary File S2

Table S1. *Systematic review summary of included studies with TGD or combined LGBTQIA+ and mixed samples.*

Intervention name	Outcomes	Author(s)	Year	Location	Study design, Data collection	Description of sample, *n*	Digital platform	Findings
**Telehealth or telemedicine interventions**								
AFFIRM Online	Depression, Coping, Stress Appraisal, Hope	Craig et al.[a]	2021	Canada	• MM: non-randomized<br>• CBT online groups	• 96 participants (46 intervention group, 50 control group: waitlist control condition)<br>• Age: intervention: M = 21.17 (SD = 4.52), control: M = 23.42 (SD = 3.41)<br>• 67.71% of total sample TGD	Zoom (videoconferencing)	Post-test intervention group:<br>• ↓ depression<br>• more likely to appraise stress as challenge, appraise resources to deal with stress as enough<br>• ↑ active coping, ↑ emotional/instrumental support, ↑ positive framing, ↑ planning, ↓ self-blame<br>• no statistically significant change in hope<br>• 95.2% of completers agreed that they learned a lot from AFFIRM Online
Project Moxie	HIV and STI testing	Sharma et al.	2019	USA	• QNT: pilot RCT<br>• HIV testing with telehealth-based counseling	• 186 participants (75 trans men, 40.3%; 33 trans women, 17.7%; 78 non-binary individuals, 41.9%)<br>• 2 age groups: 15-18 (n = 86, 46.2%), 19-24 (n = 100, 53.8%), average age: 19 years<br>• 100% of total sample TGD	VSee (videoconferencing)	Cross-sectional analysis of baseline characteristics:<br>• 6.1%-20.8% reported testing for HIV in the past year, 11.1%-33.3% reported testing for other STIs in the past year<br>• 55.4% had heard about PrEP, and 0.5% was currently on PrEP<br>• trans women less likely to report testing for HIV and other STIs

								(compared with trans men)<br>• participants treated by trans-informed HCPs three times more likely to report getting tested for HIV and other STIs than those who disagreed
	HIV and STI testing	Stephenson et al.	2020	USA	• QNT: pilot RCT<br>• HIV testing with telehealth-based counseling	• 202 participants (126 intervention HIV self-testing under supervision, 76 control HIV self-testing alone; 82 transmasculine, 36 transfeminine, 56 non-binary AFAB, 27 non-binary AMAB)<br>• 3 age groups: 15-17 (n = 66, 32.7%), 18-20 (n = 64, 32.1%), 21-24 (n = 71, 35.2%)<br>• 100% of total sample TGD	VSee (videoconferencing)	• counselors described as friendly (98-100%), knowledgeable (100%), experienced (87-100%) and professional (97-100%)<br>• participants willing to repeat intervention session (86-100%), recommend the intervention to others (68-100%), and recommend home-based HIV self-testing to others (95-100%)<br>• ↑ overall satisfaction levels with the intervention<br>• ↑ willingness to use PrEP in both groups<br>• ↑ testing for STIs in the past 3 months in the intervention (72.5%) than the control (42.9%) arm (p=0.001), but declined again by 6 months (intervention arm 19.2%, control arm 15.3%)
Telemedicine consultations in Italy	Impact of Event, Depression, Health-related Quality of Life	Gava et al.	2021	Italy	• QNT: NIS<br>• survey	• 108 participants (79 trans men, 73.1%, 29 trans women, 26.9%)	Videoconferencing (n.n.ref.)	• respondents confirmed the availability of telemedicine for endocrinological care

						• mean age: 34.3 (SD = 11.7, range = 18-61)<br>• 100% of total sample TGD		(76.8%) and mental health support (64%)<br>• 10 satisfaction scale: ↑ satisfaction 8.2 +- 2.6 for the endocrinological visit and 8.0 +- 2.4 for the psychological visit<br>• availability of telemedicine consults associated with lower impact of the COVID-19 pandemic on mental health (better IES total scores)<br>• 1/4 subjects presented a moderate to severe impact of the COVID-19 pandemic (IES score ≥ 26)<br>• 12% presented moderate to severe depressive symptoms, 18.5% mild to moderate depression (trans women ↓ than trans men)<br>• ↓ mental health-related quality of life than the general population of similar age
Unnamed online HIV counselling and testing	feasibility, key factors for choosing service options, satisfaction among MSM and trans women	Phanuphak et al.	2018	Thailand	• QNT: non-randomized<br>• online, supervised, HIV self-testing<br>• three distinct service delivery models (online, mixed, offline)	• 564 participants (465 MSM, 99 trans women)<br>• mean age: 27.9 (SD = 7.2); MSM (mean age: 28.1, SD = 7.2), trans women (mean age: 26.8, SD = 7.2)<br>• 17.55% of total sample TGD	Video chatting platform of choice of participants	Specific findings for trans people<br>• Factors associated with selecting “online pre-test counselling”: positive attitude towards HIV testing (however, prioritizing nucleic acid testing-based HIV testing decreased such chances)

								• Factors associated with selecting “online HIV testing and online post-test counseling”: being a trans woman, spending 4 to 8+ hours on social media per day, preference towards online services and home-based HIV testing<br>• Factors associated with selecting “Offline” and “Mixed” groups: being a trans woman negatively influenced mixed group selection<br>• Primary apps for conducting video calls included LINE (87.5%), Facebook messenger (20.8%), ClickDesk (8.3%), Zoom (8.3%) and Facetime (8.3%)
	linkages to HIV confirmatory testing and ART initiation	Phanuphak et al.	2020	Thailand	• QNT: non-randomized<br>• online, supervised, HIV self-testing<br>• three distinct service delivery models (online, mixed, offline)	• 564 participants (200 offline group, 156 mixed group, 208 online group; 465 MSM, 99 trans women; 45 trans women in offline, 2 in mixed, and 52 in online group)<br>• see also above	Video chatting platform of choice of participants	• 75.0% of who tested reactive (=36 people) through online, supervised, HIV self-testing subsequently accessed HIV confirmatory testing<br>• 100% of them were confirmed to have HIV infection<br>• 52.8% of these who first tested reactive by HIV self-testing successfully started ART
Unnamed telehealth intervention	intention to seek care in next month, receipt	Magnus et al.[b]	2018	USA	• MM: non-randomized pilot study	• 25 participants (trans women of color)	Video chat (Digigone), e-mail, text, or phone	• only texting, e-mailing, and phone contacts, no video chat contacts

	of care in past month				• 3-month telehealth intervention	• Age: 18-25 (n = 5, 20.0%), >25 (n = 11, 44.0%), declined to state (n = 9, 36.0%)<br>• 100% of total sample TGD		• intervention associated with ↑ odds of intention to seek transgender-specific care, having sought transgender-specific care in the last month, ↑ odds of seeking HIV-specific care<br>exit interviews after intervention:<br>• better access to and more openness to receiving health care services<br>• ↑ knowledge where to seek services<br>• participants were scared or nervous to ask about services or help, but telehealth intervention and access to the peer health consultant aided them to overcome this<br>• participant choice of mode of communication depended on preference and familiarity<br>• accessibility, knowledge base, and affability of the peer health consultants important for ability to improve access to care and overcome challenges
**Mobile health apps**								
MOTIVES	HIV knowledge and frequency of HIV testing	MacCarthy et al.[b]	2020	USA	• QNT: quasi-experimental	• 218 participants (HIV-negative MSM and trans	Mobile phone	• Both treatment arms ↑ levels of HIV prevention knowledge

					randomized pilot study<br>• Mobile Technology and Incentives (MOTIVES) to increase HIV knowledge and testing frequency<br>• weekly text messages (additionally for information plus group: win incentives by answering weekly quiz questions)	women, 2 intervention groups: 99 information only, of this 36.7% trans women, 119 information plus, of this 30.2% trans women), Comparisons (547 MSM and trans women)<br>• Age: Information Only: M = 34.8, Information Plus: M = 35.2, Comparisons: M = 33.7<br>• 12.4-36.7% of sample trans		at baseline, over time ↑ HIV prevention knowledge in information plus group<br>• Both MOTIVES intervention groups ↑ likely to test for HIV in any given 3-month interval (compared with comparison group)
	acceptability and feasibility	MacCarthy et al.	2021	USA	• QUAL: quasi-experimental randomized pilot study<br>• Mobile Technology and Incentives (MOTIVES) to increase HIV knowledge and testing frequency	• 41 study participants (n = 26 latinx sexual minority men and 15 latinx trans women) and study staff (n = 6)<br>• mean age: 37.36 (n = 41)<br>• 36.59% of total sample TGD	Mobile phone	Exit phone interviews:<br>• Acceptability: high intervention coherence<br>• participants appreciated the intervention and that it required minimal time<br>• intervention feasibility: use of technology was mentioned as both a strength and sometimes a weakness<br>• balance between programmatic and research goals: participant and study staff raised concerns about the time taken to complete research activities including

								consenting, surveys etc.
RUMAH SELA	HIV/AIDS Knowledge, Sexual Behavior, Recency of HIV Testing, Self-Esteem, Mobile App Acceptability	Garg et al.	2020	Indonesia	• QNT: non-randomized prospective intervention cohort study, • Android-based mobile app	• 168 participants: MSM (n=49), trans women (n=49), people who use drugs (n=70) • Age (years), mean (SD): MSM = 22.6 (3.3), trans women = 25.6 (3.0), people who use drugs = 23.7 (4.0) • 29.17% of total sample TGD	Mobile phone	• ↑ in comprehensive HIV-related knowledge from 22% (11/49) to 57% (28/49) among trans women (p<.001) • reduction in sexual activities without condom use from 18% (9/49) to 12% (6/49) among trans women (p=.25) • uptake of HIV testing by 49% (24/49) for trans women • measures of self-esteem increased among trans women (mean 26.5 vs mean 27.8; p=.02) • 25% (4/15) of trans women made an appointment for an HIV test through the app • acceptability of the app: majority of the participants (36/39, 74% MSM; 41/49, 84% trans women; and 47/70, 67% people who use drugs) stated that they liked the game and health facility map features of the app
Trans Women Connected	Usability testing, service utilization, goal setting, social support, PrEP knowledge	Sun et al.	2020	USA	• MM: developmental, usability non-randomized study • prototype mobile app	• 16 participants (14 trans women, 2 non-binary) • mean age: 34.5 (SD = 9.28, range = 19-52)	Mobile phone	Usability testing (phase 3) • ↑ likeability and usability (mean rating across all usability/likability questions was 5.9 out of 7)

						• 100% of total sample TGD		• ↑ multiple social support measures<br>• ↑ self-efficacy in finding LGBTQ-friendly services<br>• ↑ intention to seek online social support<br>• ↑ PrEP knowledge<br>• Positive qualitative feedback: participants felt the app was useful and attractive
Unnamed eNavigation	efficacy, HIV care continuum outcomes	Arayasirikul et al.	2020	USA	• QNT: non-randomized non-comparative<br>• text message-based, digital HIV care navigation intervention	• 120 participants (103 men, 17 trans women)<br>• mean age: 27.75 years (SD 4.07), range: 18-34 years<br>• 14.17% of total sample TGD	Mobile phone	• Baseline: 99/120 (82.5%) of the participants recently received primary HIV care; 92/120 reported taking ART (76.7%) and 65/120 having an undetectable viral load (54.2%)<br>• ↑ odds of viral suppression at 6 months compared with baseline<br>• no statistically significant additive or multiplicative interactions on comparing outcome effects over time according to intervention completion<br>• on testing for stratum-specific effects: viral suppression increased over time among those who completed the intervention
weCare	process evaluation of mobile health intervention	Tanner et al.	2020	USA	• QUAL: non-randomized non-comparative	• 32 participants: 18 intervention participants (13 cis men, 5 trans	Mobile phone	Interviews:<br>• Approach uses existing social media platforms

					• HIV care related mobile health intervention, bilingual (English + Spanish)	women), and 14 HIV clinic providers and staff<br>• Mean age (intervention participants): 25.2 (SD = 3.79; range = 20–36)<br>• 27.78% of the intervention participants trans		• HCPs therefore able to engage with participants in a more community-oriented way<br>• Bidirectional messaging allows client-centered, individualized treatment<br>• Type and frequency of intervention can be adaptively adjusted to the client's current situation<br>• Positive evaluation of approach, improvements should focus on implementation logistics, intervention content, intervention expansion
**Game-based interventions**								
Singularities	implementation success, game demand, game acceptability	Egan et al.[a]	2021	USA	• QNT: 2-arm nonblinded pilot RCT<br>• role-playing game intervention	• 240 participants (120 intervention with access to the game and a resource list, 120 control with access to the resource list only)<br>• sexual and gender minority youth, thereof 113 gender minority<br>• Age: intervention: M = 15.79, control: M =15.74, All: 15.77	Web-accessible computer	• Game demand: 55.8% of intervention participants self-reported downloading and 55.8% self-reported playing the game (lower than hypothesized thresholds).<br>• Of those that played, 68.2% reported playing an hour or greater, which exceeded hypothesis.<br>• Game-playing participants exceeded hypothesized benchmarks and reported high positive

						• 47.08% of total sample TGD		affect, low negative affect, low tension/annoyance, and high competence while playing the game.<br>• Of those who played the game, 46.2% reported a desire to play it again, and 50.8% would recommend it to friends.<br>• intervention group reported: ↓ cyberbullying victimization, ↓ binge alcohol use frequency, ↑ knowledge and greater use of online resources
Rainbow SPARX/ SPARX	acceptability	Lucassen et al.	2018	United Kingdom	• QUAL: non-randomized non-comparative<br>• computerized CBT self-help program: Rainbow SPARX	• 21 LGBT+ youth participants, 6 HCP<br>• LGBT+ young people: aged 15-22 years, mean age 17.9 years<br>• 19.05% of the LGBT+ participants trans	Web-accessible computer	• How and why LGBT+ young people use the internet to support their mental health: personal pathways (e.g., mindful videos on YouTube)<br>• HCPs concerned about safety and security (e.g., cyberbullying)<br>• Considering e-therapy like Rainbow SPARX as a useful tool: most HCPs would recommend the program when feeling down (only 8/21 LGBT+ young people recommend it for that reason)<br>• Pressure for a game-like e-therapy format to

								be as up-to-date as possible relative to commercial games
	depressive symptoms	Lucassen et al.	2020	New Zealand	• QNT: non-randomized<br>• computerized CBT self-help program: SPARX	• Finishers (n=891): baseline to last module (4 or 7): transgender (n=14), male (n=247), female (n=630)<br>• Age groups: 12-15 and 16-19; at registration: transgender (n=294), thereof 12–15 (n=131, 63.3%), 16–19 (n=76, 36.7%)<br>• 1.57% of the finishers trans	Web-accessible computer	• initial respectable recruitment of trans people, but much lost-to-follow-up<br>• trans users (n = 185) at baseline depression assessment ↑ mental health needs (69.0%)<br>• overall low completion rates at Modules 4 and 7 for all users (less than 10%)<br>• trans users reported no improvements in depressive symptoms (but for cis male and female improvements)<br>• 'look and feel' of SPARX for gender minority adolescents may in part explain the low completion rates (forced gender binary)<br>• SPARX (not Rainbow SPARX) = not trans-specific, not LGBT-specific
**Online programs or courses**								
Queer Sex Ed	feasibility, acceptability, efficacy, sexual health: sexual orientation identity and self-acceptance, sexual health knowledge, relationship variables, safer sex	Mustanski et al.	2015	USA	• MM: non-randomized non-comparative pilot study<br>• online sexual health intervention	• 202 participants: 103 male, 84 female, 14 transgender (4 AMAB, 10 AFAB), 1 N/A<br>• mean age: 17.91 years, age range: 16-20 years<br>• 6.93% of total sample TGD	Web-based software (web-accessible computer)	• majority of health outcomes (15/17) significant (p<.05)<br>• small effect sizes for sexual orientation (e.g., internalized homophobia) and relationship variables (e.g., communication skills)<br>• moderate effect sizes for safer sex (e.g.,

								contraceptive knowledge) outcomes<br>• positive attitudes toward Queer Sex Ed and high ratings for each intervention section (on average 4.2 out of 5 stars)<br>• participants reported learning more in this program than in school-based sex education and appreciated the LGBT-specific approach<br>• Low dislike for program, but disapproval of the limited amount of trans-specific information provided
QueerViBE	psychological and physical well-being	Martin[b]	2019	United Kingdom	• MM: pilot RCT<br>• online, interactive video tutorials	• 120 randomized (90 intervention, 30 waiting), 45 completed participants: intervention: 23 (13 transgender male, 10 non-binary), control: 22 (12 transgender male, 10 non-binary)<br>• mean age: intervention 18.09 years (SD=1.70), control 17.73 years (SD=1.64) (range for all: 16-21)	Video tutorials via YouTube (web-accessible computer)	• significant interactions for psychological distress with medium to large effect sizes, well-being, self-esteem, positive trans identity, appraisal of stressful events, and collective self-esteem (compared to control group)<br>• effective intervention for increasing self-reported empowerment and improving scores on measures of psychological, social and physical well-being compared to a waiting list control group<br>• perception of general online resources for

						• 100% of total sample TGD		participants: limited resources, importance of community building, education to cope with stressful situations (e.g., health care encounters, educational settings)
Unnamed MOOC	completion rate, study participants characteristics	Canavese et al.	2020	Brazil	• QNT: non-randomized non-comparative pilot study<br>• Massive Open Online Course via Moodle	• 3000 participants, 582 completers (4 trans men, 7 trans women, 14 non-binary, 119 cis men, 334 cis women, 41 other, 37 not reported)<br>• completers mean age: 29.4 (SD = 9.9), range: 15-68 years<br>• 11.34% of the completers TGD	Massive Open Online Course via Moodle (web-accessible computer)	• mixed sample of HCPs and LGBTI+ people<br>• completion rate for this MOOC (about 20%) exceeds the typical average for MOOC, which stands at about 2–10%)<br>• 63.6% (n = 370) of completers stated they had never participated in training related to health of the LGBTI+ population<br>• 48.1% (n = 280) had never interacted with LGBTI+ social movements or NGOs in their region<br>• 60.1% (n=350) were not aware of regional LGBTI+ health service actions
**Forums and online groups**								
Internet: Forums and social networks	Reasons for online interactions, types of support	Cipolletta et al.	2017	Italy	• QUAL: NIS<br>• Forums and social networks	• analysis of 122 online community conversations (Italian forums and Facebook groups involving trans people), among them 39 forum users (14 MtF; 8 FtM; 5 non-transgender	Forums, Facebook (web-accessible computer)	• 3 forums found: one for trans people only; one for LGBT; one contained a thread on transgenderism, which was moderated by a female psychologist<br>• 5 Facebook groups found: 4 for trans people and 1 for LGBT

	male; 2 non-transgender female; 10 non-identified gender, among them 4 admins, 3 moderators, 1 psychologist)<br>• online interviews with 16 users of these communities (9 MtF, 4 FtM, 1 non-identified gender, 1 male, 1 female)<br>• age of interviewees: mean = 44 years, range = 25-64 years<br>• 63.64% of total sample TGD		• total number of selected conversations: 122 (about 2440 answers and 107,935 words), comprising 46 conversations on forums and 76 conversations on Facebook groups<br>• six final categories were identified after analysis of online communities and of interviews: motivations, online help, differences between online and offline interactions, status, conflicts and professional help<br>• Findings on professional help: in case of specific medical requests tips for searching for help offline provided (e.g., list of professional names or LGBT organizations)<br>• study participants find it positive when doctors, psychologists, lawyers offer help in online communities to manage user flow and shared information

Abbreviations: *AFAB = assigned female sex at birth, AMAB = assigned male sex at birth, CBT = cognitive behavioral therapy, FtM = female to male, HCPs = health care professionals, LGBTQIA+ = lesbian, gay, bisexual, transgender, queer, intersex, asexual, + stands for the inclusive representation of all identities and expressions, MM = mixed methods study, MOOC = massive open online course, MSM = men who have sex with men, MtF = male to female, NIS = non-interventional study, QNT = quantitative study, QUAL = qualitative study, TGD = transgender and gender diverse*

[a] additional sources identified via associated published studies to the study protocols (see Supplementary File S1, Figure S1, Supplementary Material)

[b] additional articles sourced from reference lists (see Supplementary File S1, Figure S1, Supplementary Material)

Table S2. *Systematic review summary of included studies with health care professionals-only samples.*

Intervention name	Outcomes	Author(s)	Year	Location	Study design, Data collection	Description of sample, *n*	Digital platform	Findings
**Teleconsultations or e-consultations**								
VHA Transgender SCAN-ECHO / VHA e-consultation	confidence in providing care to trans veterans	Kauth et al.	2015	USA	• QNT: non-comparative pilot study (3-year feasibility program)<br>• teleconsultation and training to clinicians	• 33 participants (5 learning teams; 13 participants completed evaluation)<br>• age: n/a	VHA teleconsultation technology (videoconferencing)	• degree of confidence (0–100%) in providing care to trans veterans before and after participating in SCAN-ECHO: 39.7% increase<br>• 92.3% described didactics as ‘‘somewhat helpful’’ or ‘‘very helpful’’<br>• 76.9% reported that receiving consultation was ‘‘somewhat helpful’’ or ‘‘very helpful’’<br>• 92.3% felt they benefited from listening to other cases being discussed<br>• 76.9% found resources on the internal Web site ‘‘somewhat helpful’’ or ‘‘very helpful’’<br>• 92.3% reported that knowing experts they could contact in the future was ‘‘somewhat helpful’’ or ‘‘very helpful’’
	feasibility, typical questions from providers in e-consultation	Shipherd et al.	2016	USA	• QNT: non-comparative pilot study (3-year feasibility program)<br>• e-consultation	• 303 e-consults from 130 facilities (submitted by providers at VA	VHA teleconsultation technology (videoconferencing)	• primary questions about medications, e.g., hormone therapy (n=125), primary care medical comorbidity and screening

					hospitals, n = 177, 58%, and the rest, n = 126, 41%, were from community-based outpatient clinics)<br>• age: n/a		questions (n=97), mental health evaluations for hormone therapy and/or gender confirming surgeries (n=63), and questions about psychotherapy (n=18)<br>• secondary questions about mental health issues (n=31), medications (n=22), primary care (n=14), and psychotherapy (n=12)<br>• average amount of time spent on reviewing the chart and responding to the consult question: 78 min (63–94 min) total per consult<br>• each profession took an average of about 34 min (range = 18–58) to respond to the e-consult
providers' program experiences (qualitative), methods for improving program use (quantitative)	Blosnich et al.	2019	USA	• MM: non-randomized study (3-year feasibility program)<br>• e-consultation	• 15 interview participants (11 rural, 4 urban; 12 primary care, 3 mental health)<br>• 53 survey participants (18 rural, 35 urban; several disciplines)<br>• age: n/a	VHA teleconsultation technology (videoconferencing)	• common reasons for nonuse of program: no knowledge of program (54%), no need of program (32%), receipt of help from a colleague outside of e-consult (24%)<br>• common suggestions to improve the program use: more information about where to find e-consult in the chart,

								guidance on talking with patients about program, e-mail announcements to improve provider awareness of the program<br>• program used to establish care plans, hormone therapy recommendations, sexual and reproductive health education, surgical treatment education, patient-provider communication guidance, and second opinions<br>• suggestions for improvement: addition of concise or sectioned responses, expansion of program awareness, designation of a follow-up contact person, and increase in provider education about trans-related care
**Online trainings or courses**								
Addressing the Needs for LGBTQ-Affirming Cancer Care: A Focus on Sexual and Gender Minority Prostate Cancer Survivors	knowledge increase, satisfaction with training	Pratt-Chapman et al.	2020	USA	• QNT: non-randomized non-comparative<br>• Online training	• 204 participants (archived training, of which 77 participated in the webinar)<br>• Archived training: age group 21-29,	Webinar (web-accessible computer)	• Knowledge increase about interpersonal determinants that lead to LGBTQI health inequities (live webinar): strongly agree (71, 91.2%)<br>• Knowledge increase about unique needs of GBT prostate cancer

						n=45 (22.1%); 30-39, n=68 (33.3%); 40-49, n=40 (19.6%); 50-59 n=34 (16.7%); 60 or older, n=13 (6.4%); prefer not to answer, n=4 (2.0%); webinar: no data collected on age		patients: strongly agree (72, 94.8%)<br>• Knowledge increase about affirming and culturally sensitive strategies: strongly agree (73, 94.8%)<br>• Examples perceived as practical: strongly agree (73, 96.1%)<br>• Gained new strategies and resources for work: strongly agree (69, 90.8%)<br>• Plans for implement of new strategies and resources for work: strongly agree (63, 84.0%)<br>• Recommendation of training: strongly agree (75, 97.4%)
COLORS training	LGBT-related knowledge, attitudes, clinical practices	Seay et al.	2020	USA	• QNT: non-randomized non-comparative pilot study<br>• web-based LGBT cultural competency training	• 44 participants (oncologists), 33 (75%) completed the COLORS training<br>• mean age: 47.4 years (SD = 9.3)	Training website (web-accessible computer)	• after completing the training: ↑ LGBT-related knowledge, ↑ attitudes, ↑ clinical practices<br>• ↑ training acceptability: 82% of participants rating the training as high quality, and 97% being willing to recommend the training to a colleague
Unnamed interactive online didactic session	clinical skills, clinical preparedness, knowledge on LGBTQ health	Barrett et al.	2021	USA	• QNT: non-randomized non-comparative<br>• interactive online didactic session	• 29 participants (residents, medical students): 12 male (41%), 15 female	Videoconferencing (n.n.ref.)	• 29 attendees completed the baseline survey (15 residents and 14 medical students)<br>• 18 completed the follow-up survey (8

						(52%), 2 other/missing (7%), among them 6 LGBT-identified (21%)<br>• mean age: 29 (SD = 5)		residents and 10 medical students)<br>• baseline and follow-up scores from the clinical skills scale increased overall, in self-reported clinical preparedness, and in basic knowledge
Unnamed web-based intervention	effectiveness of intervention program, prejudices, prevalence of discrimination	Costa et al.	2016	Brazil	• QNT: non-randomized non-comparative<br>• web-based intervention	• 307 health practitioners (259 women, 84.36%; 48 men, 15.63%)<br>• average age: 34.52 years (SD = 9.40), range 18-62 years	University e-learning platform (web-accessible computer)	• ↑ pre-intervention prejudice level of HCPS (higher among those with less education, inhabiting smaller cities, with no previous education in the subject and religiosity)<br>• 68.74% were not aware of episodes of discrimination in their health services<br>• intervention was effective: percentage of participants in the lowest category of prejudice rose by 55.73 percent after the intervention (11.07%– 19.87%)

Abbreviations: *HCPs = health care professionals, LGBTQIA+ = lesbian, gay, bisexual, transgender, queer, intersex, asexual, + stands for the inclusive representation of all identities and expressions, MM = mixed methods study, QNT = quantitative study, QUAL = qualitative study, VHA = Veterans Health Administration*
