# Supplementary material for: Need for Inclusive Consideration of Transgender and Gender Diverse People in E-Health Services: A Systematic Review
Source: J Clin Med. 2022 Feb 18;11(4):1090. doi: 10.3390/jcm11041090 (PMC8880545; doi:10.3390/jcm11041090)
Supplement: Supplementary file 1 [file jcm-11-01090-s001.zip › Supplementary File S3_Search_Strategy.pdf]

Supplementary File S3: Keywords and search strategies used for the systematic review.

Concepts/Keywords

Sexual and gender minorities related terms
LGBT, sexual orientation, gender identity, lesbian, women who have sex with women, gay, men who have sex with men, bisexual, intersex, queer, polyamor*, non-monogam*, pansexual, asexual, aromantic, sexual minority, gender minority, homosexual, same-sex

Transgender related terms
transgender, transsexualism, gender dysphoria, gender incongruence, gender identity disorder, trans*, non-binary, gender diverse, genderqueer, gender fluidity, gender non-conforming, agender, genderless

E-health services related terms
eHealth, e-health, video consultation, videoconference, video service, video session, chat session, telemedicine, telehealth, teletherapy, online self-help, eTherapy, web-based therapy, internet-based intervention, digital health, e-mental health, online service, teleconsultation, eConsult, online consulting, online counseling, distance counseling, web-based, mobile health, computer-mediated communication, e-Sexual Health, mHealth, m-health, blended care, online coaching, video treatment, online treatment, blended treatment, social media health

PubMed
#1 (((((((((((((((((((LGBT) OR ("sexual orient*")) OR ("gender ident*")) OR (lesbian)) OR (“women who have sex with women”)) OR (gay)) OR (“men who have sex with men”)) OR (bisexual)) OR (intersex)) OR (queer)) OR (polyamor*)) OR ("non-monogam*")) OR ("nonmonogam*")) OR (pansexual)) OR (asexual)) OR (aromantic)) OR ("sexual minorit*")) OR ("gender minorit*")) OR (homosexual)) OR ("same-sex")
#2 ((((((((((((((transgender) OR (transsex*)) OR ("gender dysphori*")) OR ("gender incongruen*")) OR (“gender identity disorder”)) OR (trans*)) OR (nonbinary)) OR ("non-binary")) OR (“gender diverse”)) OR (genderqueer)) OR (“gender fluid*”)) OR (“gender non-conform*”)) OR (“gender nonconform*”)) OR (agender)) OR (genderless)
#3 (((((((((((((((((((((((((((((((((((“eHealth”) OR ("e-health")) OR ("video consult*")) OR (videoconference)) OR (“video service”)) OR (“video session”)) OR (“chat session”)) OR (telemedicine)) OR (telehealth)) OR (teletherapy)) OR (“online self-help”)) OR ("etherapy")) OR ("e-therapy")) OR (“web-based therapy”)) OR (“internet-based intervention”)) OR (“digital health”)) OR (“e-mental health”)) OR (“online service”)) OR (teleconsult*)) OR (“eConsult”)) OR (“online consult*”)) OR ("online counsel*")) OR (“distance counsel*”)) OR ("web-based")) OR ("mobile health")) OR ("computer-mediated communication")) OR ("e-Sexual Health")) OR ("eSexual Health")) OR ("mHealth")) OR ("m-health")) OR ("blended care")) OR ("online coaching")) OR ("video treatment")) OR ("online treatment")) OR ("blended treatment")) OR (“social media health”)
#4 ("2000/01/01"[Date - Publication] : "3000"[Date - Publication])

#1 AND #2 AND #3 AND #4

Timespan 2000/01/01 until 2021/06/30: 417 Results

Web of Science
TS=((LGBT OR "sexual orient*" OR "gender ident*" OR lesbian OR “women who have sex with women” OR gay OR “men who have sex with men” OR bisexual OR intersex OR queer OR polyamor* OR "non-monogam*" OR "nonmonogam*" OR pansexual OR asexual OR aromantic OR "sexual minorit*" OR "gender minorit*" OR homosexual OR "same-sex") AND (transgender OR transsex* OR "gender dysphori*" OR "gender incongruen*" OR “gender identity disorder” OR trans* OR nonbinary OR "non-binary" OR “gender diverse” OR genderqueer OR “gender fluid*” OR “gender non-conform*” OR “gender nonconform*” OR agender OR genderless) AND (“eHealth” OR "e-health" OR "video consult*" OR videoconference OR “video service” OR “video session” OR “chat session” OR telemedicine OR telehealth OR teletherapy OR “online self-help” OR "etherapy" OR "e-therapy" OR “web-based therapy” OR “internet-based intervention” OR “digital health” OR “e-mental health” OR “online service” OR teleconsult* OR “eConsult” OR “online consult*” OR "online counsel*" OR “distance counsel*” OR "web-based" OR "mobile health" OR "computer-mediated communication" OR "e-Sexual Health" OR "eSexual Health" OR "mHealth" OR “m-health” OR "blended care" OR "online coaching" OR "video treatment" OR "online treatment" OR "blended treatment" OR “social media health”))

Timespan 2000/01/01 until 2021/06/30: 317 Results

PubPsych
((LGBT OR "sexual orient*" OR "gender ident*" OR lesbian OR “women who have sex with women” OR gay OR “men who have sex with men” OR bisexual OR intersex OR queer OR polyamor* OR "non-monogam*" OR "nonmonogam*" OR pansexual OR asexual OR aromantic OR "sexual minorit*" OR "gender minorit*" OR homosexual OR "same-sex") AND (transgender OR transsex* OR "gender dysphori*" OR "gender incongruen*" OR “gender identity disorder” OR trans* OR nonbinary OR "non-binary" OR “gender diverse” OR genderqueer OR “gender fluid*” OR “gender non-conform*” OR “gender nonconform*” OR agender OR genderless) AND (“eHealth” OR "e-health" OR "video consult*" OR videoconference OR “video service” OR “video session” OR “chat session” OR telemedicine OR telehealth OR teletherapy OR “online self-help” OR "etherapy" OR "e-therapy" OR “web-based therapy” OR “internet-based intervention” OR “digital health” OR “e-mental health” OR “online service” OR teleconsult* OR “eConsult” OR “online consult*” OR "online counsel*" OR “distance counsel*” OR "web-based" OR "mobile health" OR "computer-mediated communication" OR "e-Sexual Health" OR "eSexual Health" OR "mHealth" OR “m-health” OR "blended care" OR "online coaching" OR "video treatment" OR "online treatment" OR "blended treatment" OR “social media health”))
PY>=2000 PY<=2021

Timespan 2000/01/01 until 2021/06/30: 416 Results
