# Supplementary material for: Need for Inclusive Consideration of Transgender and Gender Diverse People in E-Health Services: A Systematic Review
Source: J Clin Med. 2022 Feb 18;11(4):1090. doi: 10.3390/jcm11041090 (PMC8880545; doi:10.3390/jcm11041090)
Supplement: Supplementary file 1 [file jcm-11-01090-s001.zip › Supplementary File S4_Critical_Appraisal.pdf]

Supplementary File S4: Critical Appraisal of Included Studies.

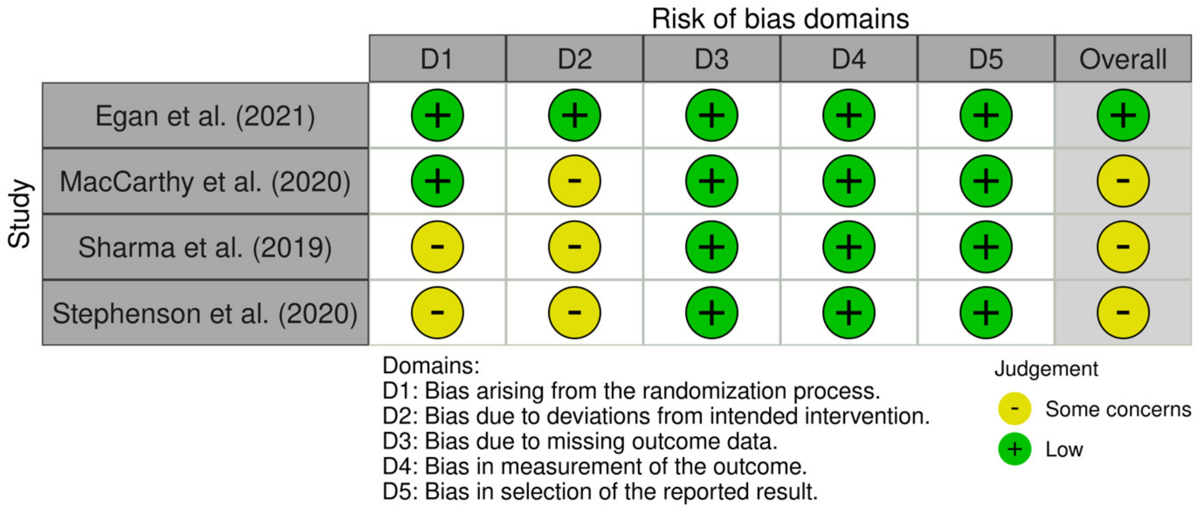

Figure S2. Results of Critical Appraisal of RCTs according to RoB 2.

Risk of bias domains

Study	D1	D2	D3	D4	D5	D6	D7	Overall
Arayasirikul et al. (2020)	-	+	?	+	X	X	+	X
Barrett et al. (2021)	-	+	?	+	X	X	+	X
Canavese et al. (2020)	-	+	?	+	X	X	+	X
Costa et al. (2016)	-	+	?	+	+	X	+	X
Garg et al. (2020)	-	+	+	+	+	-	+	-
Kauth et al. (2015)	-	+	?	+	X	X	+	X
Lucassen et al. (2020)	-	+	+	X	X	-	+	X
Phanuphak et al. (2018)	-	+	+	+	+	-	+	-
Phanuphak et al. (2020)	-	+	+	+	+	-	+	-
Pratt-Chapman et al. (2020)	-	+	?	+	X	X	+	X
Seay et al. (2020)	-	+	?	+	X	X	+	X
Shipherd et al. (2016)	-	+	?	+	+	X	+	X

Domains:
D1: Bias due to confounding.
D2: Bias due to selection of participants.
D3: Bias in classification of interventions.
D4: Bias due to deviations from intended interventions.
D5: Bias due to missing data.
D6: Bias in measurement of outcomes.
D7: Bias in selection of the reported result.

Judgement
X Serious
- Moderate
+ Low
? No information

Figure S3. Results of Critical Appraisal of Non-Randomized Quantitative Studies according to ROBINS-I.

Table S3. *Results of Critical Appraisal of Qualitative Studies.*

Study	Q1	Q2	Q3	Q4	Q5	Q6	Q7	Q8	Q9	Q10	Q11
Cipolletta et al. (2017)	Yes	Yes	Yes	Somewhat	Yes	Yes	Somewhat	Yes	Yes	Yes	Qualitative evaluation of online support networks that could promote health
Lucassen et al. (2018)	Yes	Yes	Yes	Yes	Yes	Yes	Somewhat	Yes	Yes	Yes	Qualitative evaluation of the Rainbow SPARX variant
MacCarthy et al. (2021)	Yes	Yes	Yes	Yes	Yes	Yes	Somewhat	Yes	Yes	Yes	Qualitative evaluation of the associated quantitative study (RCT)
Tanner et al. (2020)	Yes	Yes	Yes	Yes	Yes	Yes	Somewhat	Yes	Yes	Yes	Qualitative process evaluation of an implementation

Note. Optimising the value of the critical appraisal skills programme (CASP) tool for quality appraisal in qualitative evidence synthesis. According to Long, Hannah A, French, David P, Brooks, Joanna M (2020).
1. Was there a clear statement of the aims of the research?
2. Is a qualitative methodology appropriate?
3. Was the research design appropriate to address the aims of the research?
4. Are the study's theoretical underpinnings (e.g. ontological and epistemological assumptions; guiding theoretical framework(s)) clear, consistent and conceptually coherent?
5. Was the recruitment strategy appropriate to the aims of the research?
6. Was the data collected in a way that addressed the research issue?
7. Has the relationship between researcher and participants been adequately considered?
8. Have ethical issues been taken into consideration?
9. Was the data analysis sufficiently rigorous?
10. Is there a clear statement of findings?
11. How valuable is the research?
Question 11 (formerly question 10) of the CASP tool is open-ended and is therefore not largely executed in Table.

Table S4. *Results of Critical Appraisal of Mixed Methods and Remaining Studies.*

**Mixed methods studies (Questions set 1 (qualitative) + 2/3/4 (quantitative) + 5 (MM-integration) applied)**						
**Study**	**Question set**	**Q1**	**Q2**	**Q3**	**Q4**	**Q5**
Blosnich et al. (2019)	1	Yes	Yes	Yes	Yes	Yes
	4	Yes	Yes	Yes	No	Yes
	5	Yes	Yes	Yes	Yes	Yes
Craig et al. (2021)	1	Yes	Yes	Yes	Yes	Yes
	3	Yes	Yes	Yes	No	Yes
	5	Yes	Yes	Yes	Yes	Yes
Magnus et al. (2018)	1	Yes	Yes	Yes	Yes	Yes
	3	Yes	Yes	Yes	No	Yes
	5	Yes	Yes	Yes	Yes	Yes
Martin (2019)	1	Yes	Yes	Yes	Yes	Yes
	2	Yes	Yes	No	No	Yes
	5	Yes	Yes	Yes	Yes	Yes
Mustanski et al. (2015)	1	Yes	Yes	Yes	Yes	Yes
	3	Yes	Yes	Yes	No	Yes
	5	Yes	Yes	Yes	Yes	Yes
Sun et al. (2020)	1	Yes	Yes	Yes	Yes	Yes
	3	Yes	Yes	Yes	No	Yes
	5	Yes	Yes	Yes	Yes	Yes
**Cross-sectional analytic study (Question set 4 applied)**						
**Study**	**Question set**	**Q1**	**Q2**	**Q3**	**Q4**	**Q5**
Gava et al. (2021)	4	Yes	Yes	Yes	No	Yes

Note. According to Mixed Methods Appraisal Tool (MMAT), version 2018. Screening questions 1 + 2 were met for all. Question set 1 assesses the quality of the qualitative parts of study design, question set 2 assesses the RCT parts, question set 3 assesses the quantitative, non-randomized parts, questions set 4 assesses the quantitative descriptive parts, and question set 5 assesses the integration of mixed methods.
